# Tackling fraud detection with an enhanced Kepler optimization and ghost opposition-based learning

**DOI:** 10.3389/frai.2025.1710387

**Published:** 2026-01-09

**Authors:** Ria H. Egami, Amr A. Abd El-Mageed, Mona Gafar, Amr A. Abohany

**Affiliations:** 1 Department of Mathematics, College of Science and Humanity, Prince Sattam Bin Abdulaziz University, Al-Kharj, Saudi Arabia; 2 Department of Computer Science, College of Information Technology, Amman Arab University, Amman, Jordan; 3 Department of Information Systems, Sohag University, Sohag, Egypt; 4 Department of Computer Engineering and Information College of Engineering - Wadi Addawasir, Prince Sattam Bin Abdulaziz University, Al-Kharj, Saudi Arabia; 5 Faculty of Computers and Information, Damanhour University, Damanhour, Egypt

**Keywords:** feature selection, fraud detection, ghost opposition-based learning (GOBL), Kepler optimization algorithm, machine learning, metaheuristic algorithms

## Abstract

**Introduction:**

The growing prevalence of fraud and malware, fueled by increased online activity and digital transactions, has exposed the shortcomings of conventional detection systems, particularly in handling novel or obfuscated threats, class imbalance, and high-dimensional data with many irrelevant features. This underscores the need for robust and adaptive detection methodologies.

**Methods:**

This study proposes an advanced Fraud Detection (FD) methodology, BKOA-GOBL, that enhances the Binary Kepler Optimization Algorithm (BKOA) by integrating Ghost Opposition-Based Learning (GOBL) to improve Feature Selection (FS). The BKOA dynamically models gravitational attraction, planetary motion mechanics, and cyclic control to maintain a balance between exploration and exploitation. At the same time, the GOBL enhances broader search diversification and prevents early convergence, allowing the local optimum to be avoided. The Random Under-Sampling (RUS) technique is utilized to mitigate the class imbalance in fraud benchmarks.

**Results and discussion:**

Experimental validation is conducted on five real-world benchmarks, including the Australian, European, CIC-MalMem-2022, Synthetic Financial Transaction Log, and Real vs Fake Job Postings datasets, using k-Nearest Neighbors (*K*-NN) and XGBoost (Xgb-tree) classifiers. The BKOA-GOBL achieves outstanding performance, reaching classification accuracies up to 99.96% in some benchmarks and corresponding feature reduction rates up to 81.82%. Precision, recall, ROC_AUC, and F1-scores were consistently high across most benchmarks, demonstrating reliable and balanced detection. However, some challenging benchmarks—such as the Real vs Fake Job Postings dataset using *k*-NN classifier—returned lower scores (Precision = 76.14%, Recall = 66.55%, F1-score = 71.00%, and ROC_AUC = 74.15%), reflecting the difficulty of the problem. Comparative analyses against 12 recent Metaheuristic Algorithms (MHAs) and Machine Learning (ML) classifiers confirmed BKOA-GOBL's dominance in terms of accuracy and computational efficiency. Its statistical superiority is confirmed by the Wilcoxon rank-sum test, underscoring its robustness, adaptability, and effectiveness in high-dimensional fraud and malware detection tasks and real-world fraud and malware detection scenarios.

## 1 Introduction

The rise in the number of computers and mobile devices in recent years has resulted in improvements in computer network processes. Accordingly, there has been an alarming uptick in the frequency of network attacks. The ingenuity and intricacy of the attacks have been increasing, leading to a rise in the profile of network security (Adil et al., 2020; Almaiah and Almomani, 2020).

There are three primary strategies for network security: prevention, detection, and mitigation. The primary focus is on prevention. This proactive approach aims to make it hard for attacks to succeed. If prevention fails to protect the network, we use detection strategies to watch for potential threats. Finally, we have implemented mitigation strategies to ensure that devices continue to function, even during an attack. Detection strategies can be split into two types: network-based, which monitors the entire network, and host-based, which focuses on individual devices. We can also employ two methods for detection: signature-based, which identifies known threats, and anomaly-based, which detects unusual behavior (Rajadurai and Gandhi, 2022).

Intrusion Detection Systems (IDS) and malware detection are key applications that rely on network traffic classification. Host-based detection monitors a computer's internal activities, while network-based systems analyze real-time traffic logs for potential intrusions. One effective method is signature detection, which identifies known attack patterns but struggles to detect new threats (Singh A. P. et al., 2022).

Anomaly-based detection sets a threshold for expected network behavior and triggers an alarm for any deviations. It classifies data as normal or abnormal, but current Intrusion Detection Systems (IDSs) struggle with low detection accuracy and high false alarm rates (Chiba et al., 2019).

The COVID-19 pandemic has led to a significant increase in demand for online purchases of essential goods, which in turn has driven greater use of online payment methods and increased fraud and malware. With the expansion of online commerce, many enterprises have switched to credit cards for transactions. However, this increase in credit card use for online shopping has opened new avenues for criminals to exploit and steal customers' credit card information (Fanai and Abbasimehr, 2023).

Fraud in economic activities poses significant challenges across education, regulation, and business. It harms both service providers and their customers. This issue is particularly critical in the financial industry, as it affects daily financial transactions worldwide. Fraud involves using money or assets illegally for personal gain, eroding trust in financial institutions, and raising living costs. Economic fraud encompasses various harmful practices, including bank fraud, financial statement manipulation, insurance fraud, communications fraud, and illicit actions in commodity and stock markets. These fraudulent activities disrupt the global economy, push services online, and reveal recent weaknesses in the sector (Singh A. et al., 2022; Wahid et al., 2023).

Between 2000 and 2015, losses from debit and Credit Card Fraud (CCF) increased significantly. Although unauthorized transactions and fake cards accounted for only a small number of cases, they accounted for the majority of the financial losses. This issue has led both public and private sectors to invest more in advanced FD systems. These trends highlight the pressing need for robust FD strategies in the financial and e-commerce industries (Rodrigues et al., 2022).

Fraud is an illegal act, and CCF occurs when someone illegally obtains cardholder information through means such as phone calls, letters, or cyberattacks to commit financial crimes. These fraudulent activities are usually carried out using special software controlled by the perpetrator. The CCF identification process begins when a customer makes a transaction that requires verification of their credentials (Alamri and Ykhlef, 2022; Asha and KR, 2021).

The increasing prevalence of Android malware poses significant challenges for effective, efficient detection. Although traditional detection techniques, including static and dynamic analyses, have been essential for identifying malicious applications, cybercriminals have adopted evasion methods such as encryption, polymorphism, code obfuscation, and dynamic code loading to circumvent them. While dynamic analysis provides some protection against obfuscation, it struggles to scale to keep pace with the rapidly increasing volume of malicious Android applications.

As the use of Android devices increases, enhancing their security against malware threats has become critical. To address this problem, ML techniques were applied, focusing on both dynamic behaviors and static properties to detect malware. Despite this progress, there remains a need for more effective features to further enhance detection accuracy. Recently, researchers have begun exploring sonification techniques, which convert data into audio signals to reveal unique acoustic fingerprints. This innovative approach may reveal malicious features that are difficult to detect using traditional analysis methods. Additionally, sonification offers advantages such as increased processing speed, improved code coverage, and reduced resource consumption (Firdaus et al., 2018).

With the advent of big data and large datasets, in areas such as fraud and malware detection, additional problems often involve many features that individually have low discriminative power, making it difficult to achieve satisfactory classification accuracy. Classifiers tend to perform sub-optimally when faced with high-dimensional, low-quality features. Besides using highly representative features, it is also necessary to improve features through techniques such as FS and hyperparameter optimization. FS addresses the challenges of data classification in high-dimensional environments, particularly in areas such as fraud and malware detection, where many features have low discriminative power. Classifiers often perform poorly with these low-quality features, underscoring the need for efficient FS methods to identify the most relevant features and discard irrelevant or redundant ones. Traditional exhaustive search methods for determining optimal feature sets are often inefficient, prompting research into wrapper-based, biologically inspired MHAs that can simplify this process. Although these algorithms have proven effective across various applications, there is a noticeable lack of studies evaluating their performance, especially in fraud and malware detection. Ensures effective discrimination between benign and malicious transactions. FS is a method for determining the best combination of features that gives optimal results. It reduces the input feature space by removing irrelevant, redundant, or noisy features (El-Mageed et al., 2025). Using exhaustive search methods to find the best feature sets is not always practical with high-dimensional feature spaces. Biologically inspired wrapper-based MHAs have been proposed to reduce the time required to find the optimal solution (Hussien et al., 2024; El-Mageed et al., 2024; Abd El-Mageed et al., 2023). However, there is a lack of literature available on evaluating the performance of fraud and malware detection.

Taken together, financial fraud prevention, Android malware analysis, and intrusion detection all exemplify the broader challenge of high-dimensional imbalanced classification. Every domain requires models to sift through vast feature spaces dominated by benign activity, while rare but critical malicious events must be accurately identified. By framing these diverse applications under a shared methodological foundation, we highlight the continuity of challenges across domains and emphasize the importance of advanced FS and optimization strategies to improve detection accuracy and reduce false alarms.

### 1.1 Motivations

The FS plays a pivotal role in ML and data mining, especially when dealing with high-dimensional datasets, as it significantly improves classification model performance by identifying the most relevant features. The inherent complexity and large search spaces associated with FS require the use of efficient optimization algorithms (Abdel-Basset et al., 2023a). The Kepler optimization algorithm (KOA) stands out as a notable solution, which uses concepts from planetary motion to model gravitational interactions between potential features (Russell, 1964; Stephenson, 2012). This enables the KOA to skillfully balance exploration of the search space and exploitation of promising solutions (Hu et al., 2024; Houssein et al., 2024).

The most noteworthy constraint in FS is scalability, due to the challenges posed by dimensionality, which can hinder traditional search methods. The KOA excels at tackling large-scale FS issues due to its innovative structure, which enables it to navigate large search areas via typical gravitational interactions efficiently. The KOA has proven its effectiveness in tackling a variety of optimization challenges, making it a valuable tool for FS, reliably delivering high-quality solutions (Hu et al., 2024; Russell, 1964; Stephenson, 2012). The performance of KOA is characterized by fast convergence and high accuracy, which are essential for FS tasks that aim to reduce the feature set while maintaining classification accuracy (Mohamed et al., 2024). Moreover, the dynamic search mechanism in the KOA plays a pivotal role in mitigating the risk of overfitting, as it enhances feature diversity (Abdel-Basset et al., 2024).

The KOA has proven exceptionally effective in solving FS optimization problems, inspired by celestial mechanics (Abdel-Basset et al., 2023b). Its unique ability to simultaneously explore globally and exploit locally makes it a strong candidate for tackling large search spaces and high-dimensional optimization tasks. The KOA exhibits remarkable robustness in terms of solution quality and is flexible enough to adapt to various FS challenges. KOA is superior in accurately detecting a limited set of suitable attributes. But optimizing binaries and FS with a KOA requires adjustments to mitigate issues such as premature convergence and ensure ensemble diversity.

### 1.2 Contributions

This study proposes a novel BKOA-GOBL to address the FD problem by enhancing the capabilities of FS. This algorithm leverages planetary motion mechanics and integrates GOBL to escape local optima. The contribution of this study extends beyond a simple integration. The novelty of the proposed BKOA-GOBL approach lies in several methodological and conceptual enhancements that significantly improve optimization behavior, exploration-exploitation balance, and FD performance. The primary contributions of this study, which highlight the innovation of the proposed BKOA-GOBL methodology for tackling FD via FS, are summarized as follows:

- An enhanced solution updating mechanism is introduced in the proposed BKOA-GOBL, which dynamically models a gravitational-orbital updating rule that integrates planetary motion parameters (gravitational attraction, orbital velocity, and planetary distance) to refine feature subset selection. The incorporation of a cyclic control parameter and a local escaping operator boosts convergence stability and search efficiency, prevents oscillations, and improves FS accuracy–an advancement over the standard KOA.
- The class unbalance problem inherent in FD datasets is addressed through integrating the RUS technique with the proposed BKOA-GOBL methodology to achieve scalable and real-time FD. This ensures balanced training without excessive preprocessing, mitigates model bias toward the majority class, and improves the sensitivity in detecting fraudulent transactions.
- The integration of the GOBL strategy within BKOA differs from traditional opposition-based learning methods, which enhance global exploration by generating ghost-based solutions beyond the central search region using adaptive relations among the present, best, and proposed solutions. This approach enables broader and more flexible exploration, mitigates premature convergence to escape local optima, and diversifies the population, leading to more robust solutions.
- The continuous BKOA-GOBL model was redesigned for binary FS tasks via an effective threshold-based transformation and a multi-objective fitness function that simultaneously reduces the number of selected features while increasing classification accuracy–tailored for high-dimensional fraud datasets.
- Extensive evaluation across five diverse benchmarks (Australian, European, Synthetic Financial Transaction Log, CIC-MalMem-2022, and Real vs. Fake Job Postings Prediction) demonstrates the proposed BKOA-GOBL's superiority in terms of various evaluation metrics, including classification accuracy, fitness, feature reduction, precision, recall, F-score and ROC_AUC.
- Statistical validation based on the test of Wilcoxon rank-sum (5% significance level) confirms the significant superiority of BKOA-GOBL over state-of-the-art MHAs. Its consistent performance across diverse scenarios and observed improvements in convergence rate, robustness, and accuracy affirm its adaptability, robustness, and practical effectiveness for real-world FD challenges.

### 1.3 Structure

This is how the remainder of the paper is organized. Section 2 analyzes the current research in the area of fraud and malware classifications using MHTs. Section 3 explains and outlines the steps of the proposed BKOA-GOBL method to address FS issues related to fraud and malware detection; Section 4 presents the empirical findings of the recommended BKOA-GOBL and its peers, and the conclusions, in addition to problems for future investigation in Section 6.

## 2 Literature review

This section presents recent ML, DL, and metaheuristics techniques for classifying fraud and malware.

(Tarwireyi et al. 2024) investigated the use of audio features for detecting Android malware. Their study involved extracting 191 static audio features from Android micro APK datasets and evaluating fourteen different MHAs to ensure the efficiency of FS. These selected features were then used to train a light gradient-boosted classification model. The results showed that this method had high discriminatory power, with the genetic MHA achieving a significant 50.26% feature reduction and boosting classification accuracy to 99.72%.

(Toğaçar and Ergen 2024) utilized the CIC-Evasive-PDFMal2022 dataset designed by the Canadian Cybersecurity Institute, which classified PDFs into benign and malicious classes. During the preprocessing phase, parameters from text-based PDFs were transformed into 2D barcode representations. Several 2D Convolutional Neural Network (CNN) models, including ShuffleNet, ResNet18, and MobileNetV2, were trained on this data to extract distinct feature sets. The Honey Badger optimizer was employed to identify the most effective feature set, which was then classified using the softmax method, yielding a remarkable accuracy of 99.73%.

(Kaplan and Babalik 2025) employed various MHAs, including Artificial Bee Colony optimizer, Genetic Algorithm (GA), Particle Swarm Optimization (PSO), while also introducing a novel GA-PSO algorithm aimed at improving task scheduling efficiency within cloud computing, particularly under adversarial conditions such as DDoS attacks that could compromise system performance. The findings underscored the potential of advanced scheduling methods to enhance the sustainability of cloud computing while providing practical solutions to real-world security threats.

(Alashjaee 2023) proposed a new technique to improve intrusion detection (ID) called the Remora Optimization Algorithm-Levy Flight (ROA-LF). This method aims to enhance the original ROA by using Levy Flights for better performance. To test the effectiveness of ROA-LF, the researchers used various performance measures on five benchmark datasets for ID. These datasets come from data mining competitions, the ID Evaluation benchmark, and network security labs. Besides ID, ROA-LF was also applied to solve three engineering problems: pressure vessel design, three-bar truss, and cantilever beam design. Comparison showed that their proposed methodology outperformed its peers, including Particle Swarm Optimization (PSO), the Salp Swarm Algorithm (SSA), the original ROA, and the snake optimizer.

(Kale et al. 2024) developed a method that combines Black Widow Optimization (BWO) with Generative Adversarial Networks (GANs) to enhance cryptojacking detection. By optimizing features with Hybrid BWO and augmenting the dataset using GANs, they enriched the training data, resulting in a detection accuracy of 98.02%. Their approach significantly outperformed existing methods and provides a valuable framework for addressing digital security challenges.

Ghaleb et al. developed a spam detection system that combines six types of Advanced Grasshopper Optimization Algorithms (AGOA) with a Multilayer Perceptron (MLP). This system, called AGOAMLPs, effectively classifies emails as spam or not spam. Using datasets such as UK-2011 Webspam, SpamAssassin, and SpamBase, the results showed that the MLP with AGOA techniques outperformed other methods in terms of detection rate, accuracy, and reducing false alarms (Ghaleb et al., 2021).

(Ramesh et al. 2025) presented an innovative approach to cybersecurity through Enhanced Threat Intelligence for Cybersecurity Using an Ensemble of DL Models with MHAs (ETIC-EDLMHAs). It aimed to detect and effectively address network attacks. The process began with data preprocessing, which involved preparing the input data for analysis using the Word2vec model for feature extraction. In the classification phase, an ensemble of DL models was employed, notably recurrent neural networks, long short-term memory networks, and conditional variational autoencoders. Hyperparameter tuning was performed using the Wolverine optimization algorithm. Extensive simulations demonstrated that the ETIC-EDLMHAs model surpassed existing methods, achieving a remarkable accuracy of 98.51% on the CybAttT dataset.

(Mosa et al. 2024) created a framework that integrates MHAs with ML models to enhance the accuracy of fraud prediction while tackling data imbalances. They utilized 15 MHTs for FS and evaluated predictive performance using Random Forest (RF) and Support Vector Machine (SVM). Working with a Kaggle dataset containing 284,807 European card transactions, they implemented an under-sampling technique to achieve data balance. Their findings indicated that the Sailfish Optimizer, in combination with RF, achieved a classification accuracy of 97%, significantly reducing the feature set by up to 90% and improving computational efficiency.

(Prabhakaran and Nedunchelian 2023) introduced an FS method for CCF detection based on oppositional cat swarm optimization. This approach combines ML and DL techniques to improve accuracy. They employed the Oppositional Cat Swarm Optimization (OCSO) for FS. They utilized a bidirectional gated recurrent unit model for classification, along with the chaotic krill herd algorithm for hyperparameter tuning. Their research analyzed a Kaggle dataset comprising 284,807 transactions, with only 0.172% identified as fraudulent. This effectively addressed the significant class imbalance using the SMOTE technique. The results demonstrated a remarkable classification accuracy of 99.97%, surpassing traditional approaches such as Decision Trees (DTs) and RFs.

(Sorour et al. 2024) developed a CCF detection framework that utilizes the Brown Bear Optimization (BBO) algorithm to improve FS and classification accuracy while reducing dimensionality. They introduced a Binary BBO Algorithm designed to optimize feature dimensionality and used three ML classifiers–SVM, *K*-NN, and XGBoost–to detect fraudulent transactions. The framework was assessed using the Australian Credit Approval dataset and further validated on ten benchmark datasets, achieving a classification accuracy of up to 91% and reducing feature dimensionality by 67%, which significantly improved computational efficiency. Performance evaluations demonstrated that the method significantly outperformed ten other multi-hypothesis testing methods.

(Mniai et al. 2023) developed a framework designed to improve CCF detection by addressing the issue of imbalanced data and optimizing classification through FS and hyperparameter tuning. They implemented an undersampling technique to create a balanced dataset and used the Support Vector Data Description (SVDD) algorithm for classification. To enhance the hyperparameters of SVDD, they introduced a modified Polynomial Self-Learning PSO (PSLPSO) algorithm. Utilizing the Kaggle European Credit Card dataset, which consisted of 284,807 transactions with only 0.172% being fraudulent, the framework achieved a classification accuracy of 93%, outperforming models like RF, DTs, Logistic Regression (LR), and K-Nearest Neighbors (*K*-NN). This framework not only provided effective FD but also lowered computational complexity and enhanced model generalization. However, it had some drawbacks, including dataset limitations and the risk of overfitting due to the undersampling approach.

## 3 The suggested BKOA-GOBL methodology to improve FD via FS

Several interconnected stages determine this BKOA-GOBL methodology's ability to enhance FD, including managing unbalanced data, KOA-driven solution initialization and enhancement, hybridization with the GOBL strategy, and binary alteration and fitness assessment. The subsequent subsections describe these stages.

### 3.1 FD's unbalanced data addressing by RUS technique

FD datasets typically suffer from severe class unbalance, where the number of samples in non-fraud transactions overwhelmingly outnumbers the fraud ones. Typical classifiers may be biased to forecast the largest class (non-fraud transactions) as a result of this class unbalance, which could result in inadequate detection of the critical minority class (fraud transactions). Resampling techniques (Elsoud et al., 2024) are frequently employed to balance class distributions to overcome class imbalance. RUS is the most popular resampling technique chosen for this study due to its computational simplicity, effectiveness, scalability, and suitability for large-scale financial datasets. In FD, where the number of legitimate transactions can exceed that of fraudulent ones by several orders of magnitude, efficient preprocessing becomes crucial to maintaining real-time detection capabilities.

RUS (Yap et al., 2014) is a procedure to balance the dataset and equalize class distributions by randomly removing samples from the largest class (non-fraud transactions) to equal the samples in the minority class (fraud transactions). This helps reduce the bias that classifiers often develop toward the majority class in unbalanced datasets. By balancing the data, the model can better learn to detect fraudulent transactions. Although RUS may discard some useful non-fraud data, it is effective when working with large datasets where the majority class dominates. Overall, RUS enhances the system's ability to detect infrequent instances of fraud by increasing its sensitivity to the minority class. RUS is a sensible option in this case, as it minimizes the size of the largest class, which accelerates model training and eliminates unnecessary complexity. Its major advantages include:

- Computational efficiency and dataset size: the dataset utilized in this study is massive and high-dimensional. RUS substantially reduces the overall size of the training dataset, accelerating the training process of classifiers and metaheuristic optimization algorithms. This makes the training of complex models (such as BKOA-GOBL) computationally feasible and efficient without compromising the ability to identify the complex patterns of the minority class.
- Memory economy: by working on a smaller dataset, RUS minimizes storage and memory requirements, which is essential when dealing with big data environments.
- Reduction of model bias: by balancing the class proportions, RUS helps mitigate the bias of classifiers toward the dominant (non-fraud) class.
- Preventing noise and distribution shift: RUS uses only real, observed instances from the dataset, ensuring that the model is trained on genuine data points, thus mitigating the risk of introducing synthetic noise or overfitting.
- Ease of integration: RUS can be directly applied before FS or model training without introducing additional parameters or synthetic data generation, making it robust and implementation-friendly. when paired RUS with FS, yielded superior or comparable results compared to implementing hybrid sampling methods. This empirical evidence confirmed RUS as the most practical and effective balancing technique for our specific problem and model architecture.

RUS has the drawback of discarding some informative majority-class instances, which can slightly limit model generalization. To mitigate this limitation, several enhanced sampling techniques (Altalhan et al., 2025; Nguyen et al., 2024) have been developed, including Synthetic Minority Oversampling Technique (SMOTE), and Ensemble-based resampling techniques such as EasyEnsemble and BalancedBaggingClassifier. Despite these synthetic and ensemble techniques enhancing data diversity and learning balance, they require greater computational resources, increased memory usage, and more extensive parameter tuning–factors that substantially increase complexity when applied to high-dimensional and large-scale fraud datasets. Consequently, RUS was adopted in this study as a practical and computationally lightweight technique that allows the proposed BKOA-GOBL framework to focus on feature optimization and classification accuracy without incurring excessive preprocessing overhead. This choice achieves a well-balanced trade-off between computational efficiency and FD sensitivity, supporting the framework's objective of building a scalable and real-time FD system.

### 3.2 Solution initialization and enhancement using the suggested KOA

This stage is carried out using KOA (Abdel-Basset et al., 2023a), which is a physics-inspired MHA founded on Kepler's laws of planetary motion (Russell, 1964). The search region is modeled as a solar system, where the Sun represents the KOA's optimal solution and planets symbolize the KOA's potential solutions. The KOA is guided by Kepler's three rules: The planets' elliptical orbits around the Sun are stated in the first rule with a single focus. The second rule describes the variation in the Earth's speed as it revolves around the Sun: it moves quickly when it is nearer the Sun and slowly when it is farther away. The third rule states that the square of the orbital period is directly proportional to the cube of its semi-major axis, establishing a connection between a planet's orbital period and the size of its orbit. According to these rules, a planet's trajectory is influenced by its mass, position, orbital speed, and gravitational force. To properly balance exploration and exploitation during optimization, these factors form the foundation of the KOA's mathematical modeling. Theoretically, planetary locations and speeds can be predicted using Kepler's laws. The anticipated KOA's proceedings are described in depth in the subsequent subsections.

#### 3.2.1 Solution initialization

Every planet in KOA stands for a solution within the algorithm's population. A set of *N* planets, representing the population size, are created at the start of the search process to act as potential solutions in the search space. A *d*-dimensional vector, where *d* signifies the dataset's feature count, is used to represent each solution. These potential solutions are initialized randomly within their defined lower and upper boundaries. The random initialization is performed using the following formula:


$$\begin{array}{l} X_{i,j}=X_{j}^{LB}+rand\times \left(X_{j}^{UB}-X_{j}^{LB}\right). \end{array}$$ (1)

Here, *X*_*i, j*_ refers to the *i*^*th*^ initial solution for decision variables (*j*=1, 2, ..., *d*), while *rand* is a value that is created at random inside the interval [0, 1]. The terms $X_{j}^{UB}$ and $X_{j}^{LB}$ represent the upper and lower boundaries for each *j* variable, respectively. Additionally, the normal distribution is used to select the orbit period of each planet randomly. From the [0, 1], the eccentricity *e*_*i*_ of each planet's orbit is arbitrarily selected.

#### 3.2.2 Attraction of gravity computation

This step calculates the gravitational attraction between each planet and the Sun. Each planet has a unique gravitational pull, influenced by its volume and the distance between the Sun and the planet. As the planet's orbital speed increases, it gets closer to the Sun, and vice versa. The Sun's gravity as the optimal solution and planets as prospective, can be estimated as follows:


$$F_{i}^{t}=e_{i}\times \mu^{t}\times \frac{{\bar{M}}_{Best}\times {\bar{m}}_{i}}{{\bar{R}}_{i}^{2}+\epsilon }+rand_{1},$$ (2)

where *t* is the existing generation's number, and *e*_*i*_ is an arbitrary value inside [0, 1] that indicates a planet's eccentricity of orbit. To prevent the error of dividing by zero, ϵ represents a tiny value, and *rand*_1_ is an arbitrary value within [0, 1], which gives the gravity values more variation throughout the optimization process. The masses of the Sun *X*_*Best*_ and every planet *X*_*i*_ are denoted by *M*_*Best*_ and *m*_*i*_, respectively, and are determined as follows:


$$\begin{array}{l} M_{Best}={rand}_{2}\times \frac{fit\left(X_{Best}^{t}\right)-fit\left(X_{Worst}^{t}\right)}{\sum_{k=1}^{N}\left(fit\left(X_{k}^{t}\right)-fit\left(X_{Worst}^{t}\right)\right)}, \end{array}$$ (3)

$$\begin{array}{l} m_{i}=\frac{fit\left(X_{i}^{t}\right)-fit\left(X_{Worst}^{t}\right)}{\sum_{k=1}^{N}\left(fit\left(X_{k}^{t}\right)-fit\left(X_{Worst}^{t}\right)\right)}, \end{array}$$ (4)

The normalized mass values of *M*_*Best*_ and *m*_*i*_ are denoted by ${\bar{M}}_{Best}$ and ${\bar{m}}_{i}$, respectively. A random number *rand*_2_ in [0, 1] is introduced to diversify the mass values among different planets. The *t*^*th*^ generation's worst and optimal solutions are $X_{Worst}^{t}$ and $X_{Best}^{t}$, respectively, while the *k*^*th*^ solution is $X_{k}^{t}$, and the current *i*^*th*^ solution at the *t*^*th*^ generation is $X_{i}^{t}$. The worst highest and the optimal minimum fitness function values at generation *t* is given by $fit\left(X_{Worst}^{t}\right)$ and $fit\left(X_{Best}^{t}\right)$, respectively. *Ri* is the Euclidean distance among *X*_*Best*_ and *X*_*i*_, and is calculated by:


$$\begin{array}{l} R_{i}^{t}=∥X_{Best}^{t}-X_{i}^{t}{∥}_{2}=\sqrt{\overset{d}{\underset{j=1}{\sum }}{\left(X_{Best,j}^{t}-X_{i,j}^{t}\right)}^{2}}, \end{array}$$ (5)

The normalized value of *R*_*i*_ is ${\bar{R}}_{i}$. To assure the precision of the search, μ^*t*^ represents the global gravity constant, which decreases exponentially with each generation *t*. This μ^*t*^ is calculated as follows:


$$\begin{array}{l} \mu^{t}=\mu_{0}\times \exp\left(-\gamma \times \frac{t}{T_{max}}\right). \end{array}$$ (6)

where *T*_*max*_ is the allowed generations' number, γ is a constant value, and μ_0_ is a premier value.

#### 3.2.3 Planet' speed measurement

A planet's position in relation to the Sun determines its speed. For a planet near the Sun, the gravitational pull is exceedingly powerful. To avoid being drawn toward the Sun, the Earth attempts to speed away from it. Conversely, a planet's speed diminishes as the distance from the Sun increases, which decreases the Sun's gravitational influence. The mathematical formulation applied to measure the planet's speed $V_{i}^{t}$ around the Sun at the *t*^*th*^ generation is shown in Equation 7. The evaluation of planetary speed in KOA can be understood through two complementary search scenarios inspired by planetary motion around the Sun, as follows:

1. Planets close to the Sun: if the normalized distance ${\bar{R}}_{i}^{t}\leq 0.5$, the planet is considered to be near the Sun. In this scenario, due to the Sun's gravitational pull, the planet attempts to accelerate and push itself away in an effort to escape being pulled inward. In optimization terms, this condition represents a situation where a solution is in a dense or critical region of the search space, and stronger movement is needed to discover better areas. Mathematically, the speed in this scenario is influenced by either the distance between two randomly chosen solutions or the distance between the current solution and a randomly selected solution. This scenario serves to diversify the search behavior of KOA and corresponds to an exploration-oriented behavior, ensuring the algorithm does not become trapped early. However, this strategy may result in reduced speed for the planets when population diversity is limited, potentially hindering the search process. To counteract this effect and maintain adequate movement throughout the optimization, this component incorporates the distinction between the search space's upper and lower bounds, which helps maintain speed and prevents the local optimum from converging prematurely.
2. Planets away from the Sun: if ${\bar{R}}_{i}^{t}>0.5$, the planet is considered to be far from the Sun. In this scenario, the gravitational force is weaker, and the planet correspondingly reduces its speed. In optimization terms, this condition reflects that a solution is in a relatively stable and less critical region of the search space. Mathematically, depending on the distance between the present solution and a randomly selected one, the speed is decreased in this scenario. While this scenario promotes exploitation, its primary drawback is that solutions remain unchanged, which may make it harder for the algorithm to break out of the local optimum. To address this issue, the distinction between the search space's upper and lower bounds is also integrated, thereby enhancing planet mobility even in low-diversity settings.

$$V_{i}^{t}=\left\{ \begin{array}{l} \ell \times (2\times rand_{4}\times {\vec{X}}_{i}^{t}-{\vec{X}}_{b}^{t})+ℑ\times ({\vec{X}}_{a}^{t}-{\vec{X}}_{b}^{t})+(1-{\bar{R}}_{i}^{t})\\ \;\times Ϝ\times {\vec{U}}_{1}\times {\vec{rand}}_{5}\times ({\vec{X}}^{UB}-{\vec{X}}^{LB}),\;if\;{\bar{R}}_{i}^{t}\leq 0.5,\\ rand_{4}\times \zeta \times ({\vec{X}}_{a}^{t}-{\vec{X}}_{i}^{t})+(1-{\bar{R}}_{i}^{t})\times Ϝ\times U_{2}\times \vec{rand_{5}}\\ \times (rand_{3}\times {\vec{X}}^{UB}-{\vec{X}}^{LB}),\text{ otherwise}, \end{array}\right.$$ (7)

Here, ${\vec{X}}_{a}^{t}$ and ${\vec{X}}_{b}^{t}$ represent two arbitrary chosen candidate solutions (the *a*^*th*^ and *b*^*th*^) at generation *t*. The scalars *rand*_3_ and *rand*_4_ are random values drawn uniformly from the interval [0, 1], while ${\vec{rand}}_{5}$ is a random vector with elements in the same range. The parameters ℓ, ℑ, and ζ are calculated using the following expressions:


$$\begin{array}{l} \ell =\vec{U}\times M\times \zeta , \end{array}$$ (8)

$$\begin{array}{l} ℑ=\left(1-\vec{U}\right)\times \vec{M}\times \zeta , \end{array}$$ (9)

To determine the proportion of movement or step size for each planet, Equation 10 is employed.


$$\begin{array}{l} \zeta =\left[\mu^{t}\times \left(M_{Best}+m_{i}\right)\times |\frac{2}{R_{i}^{t}+\epsilon }-\frac{1}{a_{i}^{t}+\epsilon }|\right]\frac{1}{2}, \end{array}$$ (10)

$$\vec{U}=\{\begin{array}{ll} 0, & if\;{\vec{rand}}_{5}\leq {\vec{rand}}_{6},\\ 1, & \text{otherwise}, \end{array}$$ (11)

$$\begin{array}{l} M=\left({rand}_{3}\times \left(1-{rand}_{4}\right)+{rand}_{4}\right), \end{array}$$ (12)

$$\begin{array}{l} \vec{M}=\left({rand}_{3}\times \left(1-{\vec{rand}}_{5}\right)+{\vec{rand}}_{5}\right), \end{array}$$ (13)

${\vec{rand}}_{6}$ means a random vector between 0 and 1. At generation *t*, the orbital semi-major axis of planet *i* is denoted by $a_{i}^{t}$, which was calculated using:


$$\begin{array}{l} a_{i}^{t}={rand}_{3}\times {\left[T_{i}^{2}\times \frac{\mu^{t}\times \left(M_{Best}+m_{i}\right)}{4\times \pi^{2}}\right]}^{\frac{1}{3}}, \end{array}$$ (14)

The orbital period of planet *i*, denoted as *T*_*i*_, is computed as the absolute value of a randomly generated number, i.e., *T*_*i*_=|rand|. The values of the control parameters ${\vec{U}}_{1}$ and *U*_2_ are defined as follows:


$${\vec{U}}_{1}=\{\begin{array}{ll} 0, & if\;\vec{rand_{5}}\leq rand_{4},\\ 1, & \text{otherwise}, \end{array}$$ (15)

$$U_{2}=\{\begin{array}{ll} 0, & if\;rand_{3}\leq rand_{4},\\ 1, & \text{otherwise}. \end{array}$$ (16)

To lessen the possibility that planets get stuck in a local optimum, a directional flag г is introduced. This flag alters the search direction, thereby enhancing the algorithm's ability to explore the search region thoroughly. Here is a definition of the mechanism.


$$Ϝ=\{\begin{array}{ll} 1, & if\;rand_{4}\leq 0.5,\\ -1, & \text{otherwise}, \end{array}$$ (17)

#### 3.2.4 Exploration and exploitation optimization

The KOA simulates how planets move through space as they move closer and farther from the Sun by alternating between exploration and exploitation capabilities. By investigating planets farther from the Sun to detect new candidate solutions (exploration case) and intensifying the search close to the Sun to refine and improve existing solutions (exploitation case), the KOA imitates this pattern.

The following mathematical equation represents the improved solution for each planet *i* farther from the Sun ${\vec{X}}_{i}^{t+1}$ in the exploration case:


$$\begin{array}{l} {\vec{X}}_{i}^{t+1}={\vec{X}}_{i}^{t}+г\times {\vec{V}}_{i}^{t}+\left(F_{i}^{t}+|rand|\right)\times \vec{U}\times \left({\vec{X}}_{Best}^{t}-{\vec{X}}_{i}^{t}\right). \end{array}$$ (18)

In KOA, a planet's speed enables exploration when it is farther from the Sun, while the Sun's gravity encourages a planet to exploit regions near the Sun (the optimal). If the Sun represents a local optimum, the Earth can increase its speed to escape, helping the algorithm avoid a local optimum. Thus, the Sun's gravity drives exploitation, and the planet's speed ensures balanced exploration. Furthermore, to enhance the KOA's exploration and exploitation capabilities, the dynamic variation in distance between planets and the Sun is simulated. The KOA promotes exploration while planets are further from the Sun and exploitation when they are closer. This behavior is adjusted by a dynamic controlling parameter *h*–larger value enhance exploration, while smaller value favor exploitation. The stochasticity alternation between this behavior and Equation 18 strengthens the capacity of the KOA to move away from local optimum and toward global solutions. This phenomenon is represented mathematically below.


$$\begin{array}{l} {\vec{X}}_{i}^{t+1}={\vec{X}}_{i}^{t}\times {\vec{U}}_{1}+\left(1-{\vec{U}}_{1}\right)\\ \;\times \left(\frac{{\vec{X}}_{i}^{t}+\vec{X_{Best}}+{\vec{X}}_{a}^{t}}{3.0}+h\times \left(\frac{{\vec{X}}_{i}^{t}+\vec{X_{Best}}+{\vec{X}}_{a}^{t}}{3.0}-{\vec{X}}_{b}^{t}\right)\right), \end{array}$$ (19)

$$\begin{array}{l} h=\frac{1}{e^{\left(\left(a_{2}-1\right)\times {rand}_{4}+1\right)\times rand}}, \end{array}$$ (20)

$$\begin{array}{l} a_{2}=-1-1\times \left(\frac{t\%\frac{T_{max}}{TC}}{\frac{T_{max}}{TC}}\right). \end{array}$$ (21)

During the optimization process, the cyclic control parameter *a*_2_ drops by gradual from −1 to −2 for *TC* cycles.

### 3.3 GOBL strategy incorporation

To enhance the KOA's capacity to escape local optima, this paper incorporates a GOBL strategy. Unlike traditional opposition-based learning methods (Tizhoosh, 2005; Mahdavi et al., 2018), which rely on a fixed central point within the search space and generate opposite solutions confined to the midpoint region, GOBL introduces greater flexibility and spatial diversity. Traditional opposition-based learning is centered around a fixed midpoint within the search space. In this framework, opposite solutions are generated based on a static rule that reflects the current position around the center of the exploration range. As a result, the newly generated solutions tend to cluster near this midpoint, and their spatial extent typically does not surpass the distance between the present solution and the central point, making it challenging for the algorithm to investigate regions distant from the central area where the global optimum is located and may struggle to escape local optima.

In contrast, GOBL dynamically combines information from the present individual, a proposed individual, and the best individual found so far to replace poor proposed positions with newly generated ghost possible solutions. These ghost solutions are designed to extend beyond the conventional bounds set by the midpoint, thereby enabling broader and more adaptive exploration and increasing the chances of escaping local optima, especially when the global optimum is far from the search center. To better illustrate the GOBL strategy, suppose a space with two dimensions defined by the X-axis and Y-axis. The X-axis defines the search boundaries [*LB, UB*]. Within this space, let *X*_*new*_ denote the position of a newly generated possible solution with a height *h*_*new*_. The best solution discovered is projected onto the X-axis at *X*_*Best*_ position with a *h*_*Best*_ height. Also, the present possible solution has a projection *X*_*i*_ with height *h*_*i*_. Using these reference points, the position of the ghost *x*_*i*_ with height *h*_*i*_ is calculated, as follows:


$$\begin{array}{l} x_{i}=X_{new}-X_{i}+X_{Best}. \end{array}$$ (22)

Let *P*_*i*_=(*x*_*i*_, *h*_*i*_) represent the ghost position, where *x*_*i*_ represents the X-axis projection and *h*_*i*_ is the height. In this context, the Y-axis is used metaphorically as a convex lens to simulate optical imaging. When *P*_*i*_ passes through the lens, it produces a genuine image $P_{i}^{*}$=$\left(x_{i}^{*},h_{i}^{*}\right)$, where $x_{i}^{*}$ corresponds to the opposite solution of *x*_*i*_. Hence, the relationship between the ghost position and its genuine image is defined by:


$$\begin{array}{l} k_{i}=\frac{h_{i}}{h_{i}^{*}}=\frac{\frac{\left(UB+LB\right)}{2}-x_{i}}{x_{i}^{*}-\frac{\left(UB+LB\right)}{2}}. \end{array}$$ (23)

As a result, the GOBL calculation can be derived from the previous equation to generate opposition-based solutions that go beyond traditional midpoint reflections, as follows:


$$\begin{array}{l} x_{i}^{*}=\frac{\left(UB+LB\right)}{2}+\frac{\left(UB+LB\right)}{2k}-\frac{x_{i}}{k}. \end{array}$$ (24)

### 3.4 Binary alteration and assessment of continuous solution

In FS, the goal is to lessen the number of features while retaining classification effectiveness. Achieving this requires careful selection of the most relevant features and discarding those that negatively impact the classification accuracy. In binary optimization, FS problems require encoding solution representations as binary vectors. Since the KOA operates in a continuous domain, which is incompatible with binary FS problems, it must be adapted by altering the continuous values to a binary format. Each solution is represented as a one-dimensional binary vector, where 1 mentions a picked feature and 0 mentions exclusion. The transformation of a continuous solution $X_{i}^{t}$ into its binary counterpart ${\left(X_{i}^{t}\right)}_{binary}$, using a random threshold *thr*_*rand*_ in [0, 1], is defined by the following rule:


$${\left(X_{i}^{t}\right)}_{binary}=\{\begin{array}{ll} 0 & \text{ if }X_{i}^{t}<thr_{rand},\\ 1 & \text{ otherwise}. \end{array}$$ (25)

*thr*_*rand*_ should be chosen with consideration of the problem context, as this directly influences the FS behavior. Different problem scenarios may require different threshold settings to achieve optimal performance.

To assess a solution's quality, two conflicting objectives must be balanced: increasing the accuracy of classification and minimizing the number of chosen features. While high accuracy ensures reliable predictive performance and utility of models, aggressive feature reduction can lead to performance degradation. Therefore, a well-balanced fitness function is essential. It incorporates both the size of the feature subset and the accuracy of classification, and is described mathematically as:


$$\begin{array}{l} \text{Fitness}=w_{1}\times \left(1-\text{classification accuracy}\right)+w_{2}\times \frac{\left|d^{*}\right|}{\left|D\right|}, \end{array}$$ (26)

where (1 − classification accuracy) denotes the misclassification error rate, *D* refers to the features' count, and d^*^ means the features chosen's count. *w*_1_ and *w*_2_ signify the contribution from the accuracy and the cardinality of the feature sets, respectively, with *w*_1_∈[0, 1], and *w*_2_=1−*w*_1_.

**Figure 1:**
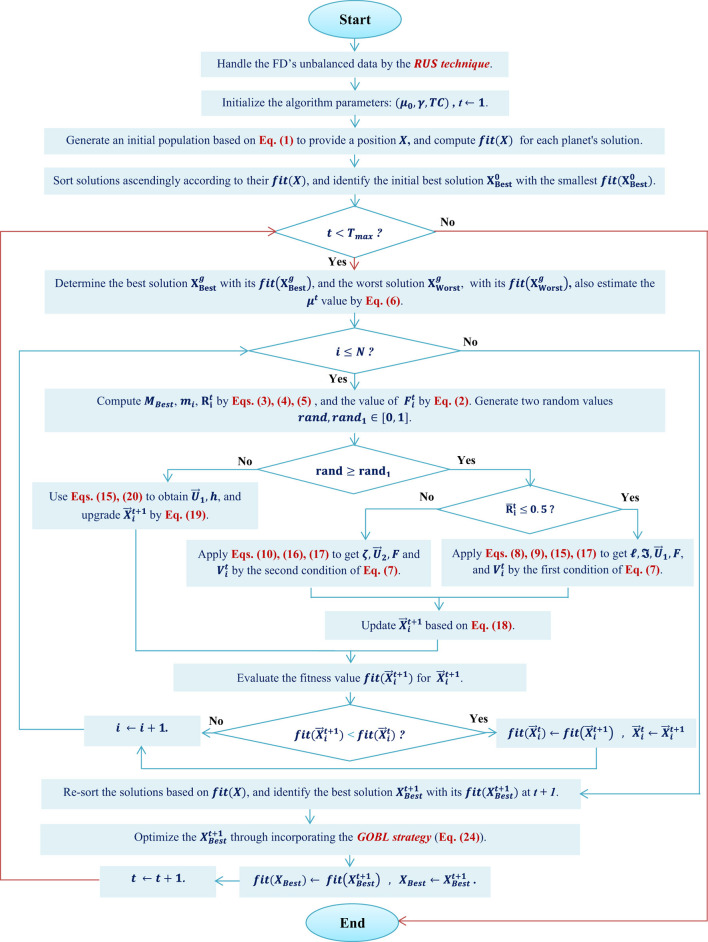
Flowchart of the suggested BKOA-GOBL methodology.

After presenting the fundamental stages of the suggested BKOA-GOBL in the previous subsections, the BKOA-GOBL's pseudo-code is summarized in Algorithm 1. Additionally, the whole process and key stages of the BKOA-GOBL are also illustrated in the flowchart in Figure 1.

Algorithm 1 **The recommended BKOA-GOBL methodology.**

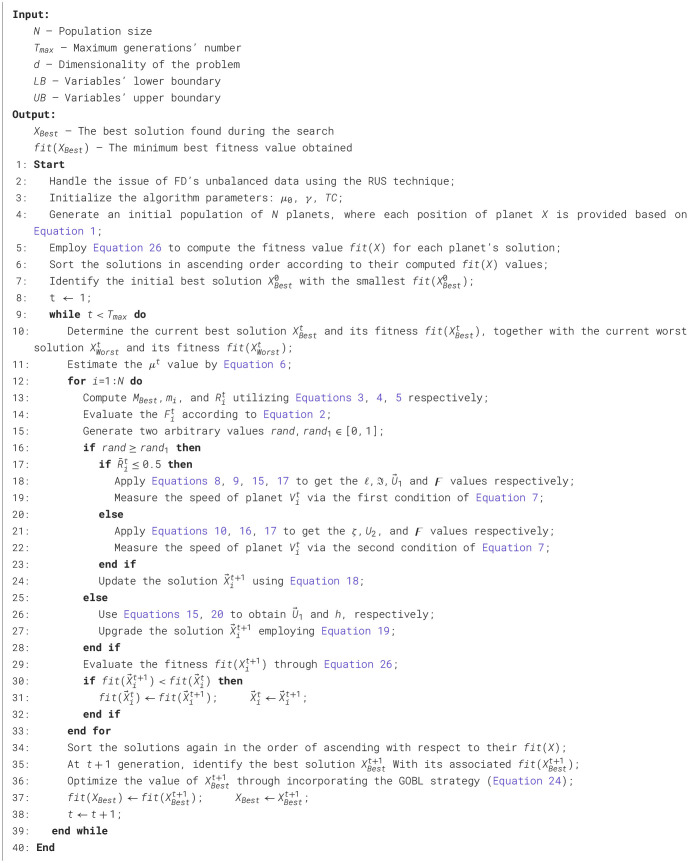

### 3.5 Computational complexity of the BKOA-GOBL methodology

#### 3.5.1 Time computational complexity of the BKOA-GOBL methodology

The time computational complexity of the proposed BKOA-GOBL methodology can be evaluated by analyzing its core stages that collectively contribute to its performance improvement in FD. These stages include addressing class imbalance using the RUS technique, generating and refining solutions via the KOA, incorporating the GOBL strategy, and performing binary alterations on solutions, as well as evaluating the fitness function. The total time computational complexity, expressed in big-O notation, *O*_*time*_(*BKOA*−*GOBL*), is derived as follows:

- RUS technique: balances the dataset by randomly removing samples from the majority class to equalize the number of minority and majority samples. This process operates linearly with respect to the number of instances *S* in the dataset, resulting in a time complexity of *O*_*time*_(*S*).
- Solution generation and refinement: Generates an initial population of *N* candidate solutions, each represented in a *d*-dimensional search space. The time complexity of this process is *O*_*time*_(*N*×*d*). After that, each candidate's position is updated iteratively based on gravitational and orbital dynamics across *G*_*max*_ generations. The time complexity of this iterative update is *O*_*time*_(*G*_*max*_×*N*×*d*).
- GOBL strategy: generates ghost-based opposition solutions to replace inferior candidates and maintain diversity. The computational cost of this step is proportional to both the population size and the problem dimension, resulting in a time complexity of *O*_*time*_(*N*×*d*).
- Binary alteration and fitness function assessment: converts continuous feature representations into binary form during each iteration for all individuals to adapt the continuous KOA for discrete FS. This step has a time complexity of *O*_*time*_(*G*_*max*_×*N*×*d*). Then, the classification-based fitness for each solution has been computed at every iteration. Assuming each fitness computation depends primarily on model accuracy using *M* classifier evaluations, this step has a time complexity of *O*_*time*_(*G*_*max*_×*N*×*M*), which simplifies to *O*_*time*_(*G*_*max*_×*N*) when *M* is constant.

Where *N* means the number of individuals in the population, *G*_*max*_ is the maximum iterations allowed, and *d* identifies the dimensionality of the problem space. After that, the overall time computational complexity of the BKOA-GOBL can be determined by combining all stages as follows:


$$\begin{array}{c} O_{time}\left(BKOA-GOBL\right)\\ =O_{time}\left(\text{RUS technique}\right)\\ +\;O_{time}\left(\text{Solution generation and refinement}\right)+\\ +\;O_{time}\left(\text{GOBL strategy}\right)\\ +\;O_{time}\left(\text{Binary alteration and fitness function assessment}\right). \end{array}$$

$$\begin{array}{c} O_{time}\left(BKOA-GOBL\right)\\ =\;O_{time}\left(S\right)+O_{time}\left(N\times d\right)\\ +\;O_{time}\left(G_{max}\times N\times d\right)+O_{time}\left(N\times d\right)\\ +\;O_{time}\left(G_{max}\times N\times d\right)+O_{time}\left(G_{max}\times N\right). \end{array}$$

After simplification, the overall time computational complexity is dominated by the iterative BKOA-GOBL-driven solution update and binary conversion processes, resulting in:


$$\begin{array}{c} O_{time}(G_{max}\times N\times d). \end{array}$$

This time complexity *O*_*time*_(*G*_*max*_×*N*×*d*) is consistent with other population-based metaheuristic algorithms used for FS. While the inclusion of GOBL and binary alteration increases computational demand, these additions significantly enhance exploration and exploitation balance, reducing the risk of premature convergence and improving FS quality. The trade-off between computational cost and detection accuracy is justified, as BKOA-GOBL achieves superior convergence, robustness, and scalability across high-dimensional datasets for FD. Additionally, the algorithm can benefit from parallel and distributed implementations, where the evaluation of candidate solutions can be executed concurrently, effectively mitigating computational overhead in large-scale applications.

#### 3.5.2 Space computational complexity of the BKOA-GOBL methodology

The space computational complexity reflects the memory usage or storage space required for the BKOA-GOBL algorithm to handle a problem as the input size grows. It includes the memory required to store all input variables, internal vectors, temporary structures, and auxiliary states used during the optimization process. The following analysis divides the total required memory into two main components:

- Memory space complexity of input parameters: this refers to the memory needed to store the algorithm's input parameters to operate. The proposed BKOA-GOBL framework (as shown in Algorithm 1) utilizes eight input variables: *N*, *T*_max_, *d*, μ_0_, γ, *TC*, *LB*, and *UB*. Each variable is stored as a single numerical value requiring 4 bytes of memory. Thus, the total memory footprint for the input variables is: (8 × 4 = 32 bytes), and therefore the input values space complexity contributes only constant memory.
- Memory space complexity of contributory parameters: this refers to the additional temporary storage required by the algorithm during optimization for internal computations. It consists of the following components:

- Population vector *X*: the BKOA-GOBL maintains a population of *N* continuous candidate solutions, each of dimension *d*. Each solution is a floating-point value requires 4 bytes; therefore, the memory space required is: (4 × *N*×*d*) bytes. This contributes linear space complexity in terms of *N*×*d*.
- Binary population vector *X*_*binary*_: After binarization, each candidate solution is represented as a binary vector of dimension *d*, consuming 1 byte per entry, but approximated by standard 4-byte allocation for uniformity. Thus, the memory space for binary vectors: (4 × *N*×*d*) bytes, which is linear in *N*×*d*.
- Fitness values and scalar variables: the algorithm stores the fitness of all individuals (*fit*(*X*_*i*_), *fit*(*X*_*k*_)), optimal and worst solutions (*fit*(*X*_*Best*_), *fit*(*X*_*Worst*_)), orbital quantities (*M*_*Best*_, *m*_*i*_, *F*_*i*_, *R*_*i*_, *a*_*i*_, ϵ, *T*_*i*_, *e*_*i*_, *V*_*i*_, ℑ, г, ζ, ℓ, M, $\vec{U}$, ${\vec{U}}_{1}$, *U*_2_), control parameters (μ, μ_0_, γ, *T*_*max*_, *LB*, *UB*, *h*, *TC*, *a*_2_), and random coefficients (*rand*, *rand*_1_, *rand*_2_, *rand*_3_, *rand*_4_, ${\vec{rand}}_{5}$, ${\vec{rand}}_{6}$). In total, the algorithm uses 37 such scalar variables, each requiring 4 bytes: (37 × 4 bytes = 148 bytes), which corresponds to constant space.
- Population vectors: the population in the algorithm consists of eight vectors: *X*_*i*_, *X*_*k*_, *X*_*Best*_, *X*_*Worst*_, *X*_*a*_, *X*_*b*_, *X*_*new*_, $x_{i}^{*}$. Each vector has a dimensionality of *d*. Since every position requires 4 bytes of memory, each vector occupies (4 × *d*) bytes. Therefore, the total complexity of the memory space required for all eight vectors is: 8 × 4 × *d* bytes = 32 × *d* bytes. This results in a linear space complexity with respect to dimensionality *d*.

Thus, the total complexity of memory space for the previous contributory parameters is:


$$\begin{array}{l} \left(4\times N\times d\right)+\left(4\times N\times d\right)+148+\left(32\times d\right)bytes. \end{array}$$

Putting everything together, the total memory space complexity of the BKOA-GOBL methodology is:


$$\begin{array}{c} \text{Memory space complexity (BKOA-GOBL)}\\ =\;\text{Space complexity of input parameters}\\ \;+\;\text{Space complexity of contributory parameters}\\ =\;32+\left(\left(4\times N\times d\right)+\left(4\times N\times d\right)+148+\left(32\times d\right)\right)\text{bytes}. \end{array}$$

Ignoring all constants, the big-O notation *O*_*space*_(*BKOA*−*GOBL*) for the total memory space complexity of the BKOA-GOBL becomes:


$$\begin{array}{c} O_{space}(BKOA-GOBL)\\ =\;O_{space}(\text{Space complexity of input parameters})\\ +\;O_{space}(\text{Space complexity of contributory parameters})\\ =\;O_{space}(1)+(O_{space}(N\times d)+O_{space}(N\times d)\\ +\;O_{space}(1)+O_{space}(d))=O_{space}(N\times d). \end{array}$$

Therefore, the overall space computational complexity of the BKOA-GOBL methodology is:


$$\begin{array}{c} O_{space}(N\times d). \end{array}$$

## 4 Experimental results and analysis

The experimental results for the proposed BKOA-GOBL methodology, in comparison to various alternative algorithms, are described in detail in this section. The presented technique is verified utilizing three distinct benchmark datasets from multiple sources. The average and Standard Deviation (STD) of the evaluation metrics were estimated and presented. Information regarding the benchmark datasets and the parameters for MHTs can be found in Sections 4.1, 4.2, respectively. Performance metrics are explained in Subsection 4.3. The results of the recommended BKOA-GOBL via *k*-NN, and Xgb-tree classifiers are discussed in Subsection 4.4. The findings of the BKOA-GOBL against its peers are studied in Sections 4.6, 4.7. The convergence graphs are also depicted in Section 4.8. Finally, Wilcoxon's test determines the differences in the values of fitness between the proposed BKOA-GOBL and its competitors.

### 4.1 Benchmarks description

In this section, we examine five publicly accessible datasets that are frequently utilized in the creation and assessment of classification models for FD, cybersecurity, and financial decision-making. These datasets encompass a range of domains, including credit approval, transaction fraud, malware analysis, economic simulation, and employment fraud. Each dataset is distinguished by its complexity of features, number of records, class distribution, domain specificity, and availability. Table 1 provides a summary of the key attributes of each dataset, along with direct access links for reproducibility and further investigation.

**Table 1:** Comparison of five datasets utilized in this study.

**ID**	**Dataset name**	**#Features**	**#Records**	**#Classes (names)**	**Balance**	**Domain**	**Source**
1	Australian	14 (6 numeric, 8 categorical)	690	2 (approved, not approved)	52% approved, 48% denied	Credit approval decision-making	UCI Repository
2	European	30 (PCA: V1–V28, time, amount)	284,807	2 (legitimate, fraud)	Extremely imbalanced (0.17% fraud)	Financial transaction FD	Kaggle (2013)
3	Synthetic financial transaction log	11 (step, type, amount, balances, etc.)	100,000+	2 (legitimate, fraud)	Highly imbalanced (0.1%–0.2% fraud)	Mobile money fraud simulation	Kaggle (2016)
4	CIC-MalMem-2022	55–56	58,596	4 (Benign, Trojan, Spyware, Ransomware)	Balanced binary; malware families 16%–17% each	Cybersecurity/obfuscated malware detection	Canadian Institute for Cybersecurity (CIC), University of New Brunswick (UNB) (2022)
5	Real vs. Fake Job Postings Prediction	18 (text + metadata fields)	17,880	2 (real, fake)	4.5% fake (800 fraudulent)	Employment scam/job FD	Kaggle (2020)

### 4.2 Parameters configuration

The BKOA-GOBL was assessed alongside several binary versions of different MHTs, which included the original BKOA and ten recent MHTs. Each algorithm was tested thirty times per dataset to account for variability, and average performance metrics were provided for equitable comparisons. To achieve equity, all MHTs were governed by a 10 size of population and a limit of 100 generations. The attributes of the datasets indicated the scale of the problem, while the continuous search domain was set to [−1, 1] to create a broad yet controlled search space.

A 10-fold cross-validation method was employed for evaluating the generalizability and robustness of the BKOA-GOBL and its competitors. The datasets were split into 80% training and 20% testing subsets. The training subset was utilized to fine-tune the classifiers, while the testing subset was used to assess the effectiveness of the chosen features. Parameter configurations for each technique adhered to the original specifications established in foundational studies, with a summary presented in Table 2. The experiments were conducted in a Python environment on a high-performance computing system equipped with 256 GB of RAM and a Dual Intel Xeon Gold 5115 CPU, running on Microsoft Windows Server 2022.

**Table 2:** Parameter settings for used optimizers.

**Optimizer**	**Coefficients**
All MHTS	Runs' number = 30
	Generations' number *T*_*max*_ = 100
	Population Size *N* = 10
	Dimensions *d* = The number of features in the employed
	benchmark
BKOA-GOBL	Premier value μ_0_ = 0.1
	Parameter γ = 15
	Cycles' number during the entire optimization process *TC* = 3
	Minimum value of the decision variables =-1
	Maximum value of the decision variables =1
Binary Meerkat Optimization Algorithm (BMOA)	*Sentry* = 0.3
	*P* = 0.5
	β=1.5
	*r* = 1
Binary Salp Swarm Optimizer (BSSO)	Safety threshold *ST* = 0.8
	Scroungers' number *SD* = 0.1^*^N
	Producers' number *PD* = 0.2^*^N
Binary Aquila Optimizer (BAO)	Search cycles' number 𝔯_1_ = 10
	ω=0.005
	*U*=0.00565
	Aquila's flying slope _2_∈[2, 0]
	Adjustment coefficients for exploitation phase δ = 0.1,
	and α = 0.1
	Aquila's arbitrary motions _1_∈[−1, 1]
Binary Atom Search Optimization (BASO)	Depth weight α = 50
	Multiplier weight β = 0.2
Binary Harris Hawks Optimization (BHHO)	Rabbit energy *E*∈[−1, 1]
Binary Henry Gas Solubility Optimization (BHGSO)	β = 0.1=α*and*K=1
	Clusters' number is 1
	*l*_1_ = 5*E*−03, *l*_2_ = 1*E*+02, and *l*_3_ = 1*E*−02
Binary Bat Algorithm (BBA)	Pulse emission rate *r* = 0.95
	Loudness *A* = 0.8
	Minimum and Maximum pulse frequencies = 0, 10
Binary Sailfish Optimizer (BSFO)	*A* = 1
	Ratio between sailfish and sardines *pp* = 0.1
	ε = 0.0001
Binary Grasshopper Optimization Algorithm (BGOA)	*C*_max_ = 1 and *C*_min_ = 0.00004
Binary Sparrow Search Algorithm (BPSO)	Acceleration coefficients (*c*_1_ = *c*_2_ = 1.2)
	Inertia weight (ω_min_ = 0.4, ω_max_ = 0.9)

Table 3 presents the main coefficients of the ML classifiers employed in this study.

**Table 3:** The primary parameters of the ML models.

**Classifier**	**Parameters**
Xgb-Tree	Boosting rounds' number *nrounds* = 100
	Min loss reduction *gamma* = 0
	Max depth *max*_*depth* = 3
	Learning rate *eta* = 0.4
	Min sum of instance weight *min*_*child*_*weight* = 1
	Sub-sample ratio of learning *sub*_*sample* = 0.75
	Sub-sample ratio of columns *colsample*_*bytree* = 0.8
*k*-NN	Euclidean distance *k* = 5

### 4.3 Evaluation measures

We utilize a wide range of evaluation measures to measure the efficacy of the suggested BKOA-GOBL for fraud and malware detection. These measures are essential for evaluating the model's predictive ability, stability, and effectiveness.

- Accuracy (AC): accuracy evaluates how correct the model is by determining the ratio of instances that have been classified correctly, as in Equation 27.

$$\begin{array}{l} ACC=\frac{TN+TP}{TN+TP+FN+FP} \end{array}$$ (27)

where:

- True positives (TP): unauthorized transactions accurately recognized as Fraud.
- True negatives (TN): trustworthy transactions accurately recognized as authentic.
- False positives (FP): trustworthy transactions inaccurately recognized as fraud.
- False negatives (FN): unauthorized transactions inaccurately recognized as trustworthy.

An increased accuracy reflects improved model performance.

- Fitness function: the fitness function of the KOA optimizer estimated the performance of the model by finding a balance between classification accuracy and FS.
- Size of chosen attributes: this metric reflects the total number of features retained after the SBO algorithm performs FS. Reducing the number of features while maintaining high accuracy improves the model's efficiency and interpretability.
- This measure estimates the size of features contained after applying the KOA for FS. Minimizing the size of selected features while preserving increased accuracy enhances both the efficiency and interpretability of the model.
- Precision (P): precision (De Medeiros et al., 2007) measures the percentage of accurately identified fraudulent transactions out of all transactions that were classified as fraudulent, as in Equation 28.

$$\begin{array}{l} P=\frac{TP}{TP+FP} \end{array}$$ (28)

- Recall (R): recall (Amigó et al., 2009) estimates the ability of the model to identify fraud transactions accurately, as in Equation 29.

$$\begin{array}{l} R=\frac{TP}{TP+FN} \end{array}$$ (29)

- F1-Score: the F1-score (Amigó et al., 2011) is the harmonic average of recall and precision, providing a specific measure that balances both, as in Equation 30.

$$\begin{array}{l} F1=2\times \frac{P\times R}{P+R} \end{array}$$ (30)

In the following subsections, we will thoroughly review and investigate the experimental outcomes, highlighting the significant results in bold.

### 4.4 Empirical outcomes of two ML models (Xgb-tree, and *K*-NN) and the suggested BKOA-GOBL

This section compares the results of the *K*-NN and Xgb-tree models with the proposed BKOA-GOBL. It focuses on assessing their effectiveness by examining average classification accuracy and the average number of features selected, enabling us to gauge the impact of the BKOA-GOBL method.

Table 4 illustrates the performance metrics for the proposed BKOA-GOBL alongside the primary *K*-NN, focusing on mean accuracy and the size of the selected features. As depicted in Table 4, the proposed BKOA-GOBL combined with *K*-NN has significantly enhanced classification accuracy across five benchmark datasets, achieving an increase of 11.52% in the Australian dataset, 44.76% in the European dataset, 3.56% in the Synthetic Financial Transaction Log dataset, 31.37% in the Real vs. Fake Job Postings Prediction dataset, and a slight improvement of 0.05% in the CIC-MalMem-2022 dataset. Furthermore, the BKOA-GOBL method has led to a reduction in the number of features selected from the benchmark datasets, with decrease rates of 58.57% in the Australian dataset, 62.77% in the European dataset, 80.00% in the CIC-MalMem-2022 dataset, 81.82% in the Synthetic Financial Transaction Log dataset, and 48.96% in the Real vs. Fake Job Postings Prediction dataset.

**Table 4:** Outcomes of the basic *K*-NN classifier and the suggested BKOA-GOBL concerning mean accuracy and size of picked features.

**Dataset**	**Classification accuracy**			**Size of chosen features (decrease rate** = $\frac{\text{Original Features}-\text{Selected Features}}{\text{Original Features}}\times 100\%$**)**		
	*K* **-NN**	**BKOA-GOBL**	**Increase rate (%)**	*K* **-NN**	**BKOA-GOBL**	**Decrease rate (%)**
Australian	0.8116	**0.9051**	11.52%	14.00	**05.80**	58.57%
European	0.6548	**0.9523**	44.76%	30.00	**10.27**	62.77%
Synthetic financial transaction log	0.9512	**0.9851**	03.56%	11.00	**02.00**	81.82%
CIC-MalMem-2022	0.9991	**0.9996**	0.0500%%	55.00	**11.00**	80.00%
Real vs. Fake Job Postings Prediction	0.5642	**0.7412**	31.37%	1426	**727.83**	48.96%

Bold values indicate the best-performing results for each evaluation measure.

Additionally, Table 5 illustrates the performance metrics for the proposed BKOA-GOBL alongside the primary Xgb-tree, focusing on mean accuracy and the size of the selected features. As shown in Table 5, the suggested BKOA-GOBL combined with Xgb-tree has significantly improved accuracy across five benchmark datasets, achieving an increase of 05.94% in the Australian dataset, 02.70% in the European dataset, 4.08% in the Real vs. Fake Job Postings Prediction dataset, and a slight improvement of 0.0600% in the CIC-MalMem-2022 dataset and 0.0504% in the Synthetic Financial Transaction Log dataset. Furthermore, the BKOA-GOBL method has led to a reduction in the number of features selected from the benchmark datasets, with decrease rates of 55.50% in the Australian dataset, 53.33% in the European dataset, 83.45% in the CIC-MalMem-2022 dataset, 60.91% in the Synthetic Financial Transaction Log dataset, and 50.22% in the Real vs. Fake Job Postings Prediction dataset.

**Table 5:** Outcomes of the basic Xgb-tree classifier and the suggested BKOA-GOBL concerning mean accuracy and size of picked features.

**Benchmark**	**Accuracy**			**Size of chosen features (decrease rate** = $\frac{\text{Original Features}-\text{Selected Features}}{\text{Original Features}}\times 100\%$**)**		
	**Xgb-tree**	**BKOA-GOBL**	**Increase rate (%)**	**BKOA-GOBL**	**BSBO–MUT**	**Decrease rate (%)**
Australian	0.8623	**0.9135**	05.94%	14.00	**06.27**	55.50%
European	0.9239	**0.9489**	02.70%	30.00	**12.10**	53.33%
Synthetic financial transaction log	0.9912	**0.9917**	0.0504%	11.00	**04.30**	60.91%
CIC-MalMem-2022	0.9991	**0.9997**	0.0600%	55.00	**09.10**	83.45%
Real vs. Fake Job Postings Prediction	0.7838	**0.8158**	4.08%	1426	**709.83**	50.22%

Bold values indicate the best-performing results for each evaluation measure.

Finally, the BKOA-GOBL method surpassed the basic ML models, *K*-NN, and XGB-Tree in terms of mean accuracy and the number of selected attributes across the five datasets. This demonstrates its potential effectiveness for FS in comparison to these basic ML models.

### 4.5 Comparative evaluation of the suggested BKOA-GOBL under various resampling techniques

To further validate the robustness and adaptability of the proposed BKOA-GOBL framework, additional experiments were performed using several resampling techniques to handle the class imbalance challenge commonly observed in FD datasets. Specifically, the RUS technique adopted in the primary BKOA-GOBL framework was compared with two Ensemble-based resampling techniques, namely EasyEnsemble and BalancedBaggingClassifier. Each resampling method was applied at the preprocessing stage before FS and classification to ensure a fair comparative assessment across all experiments.

Table 6 presents the comparative performance metrics obtained across the examined datasets, including accuracy, fitness score, number of selected features, precision, recall, F1-score, and ROC_AUC. The results clearly show that RUS delivers the strongest and most consistent performance across the majority of the evaluation measures. When paired with the BKOA-GOBL optimization framework, RUS achieves the highest or near-highest accuracy and ROC_AUC values on most datasets, while also generating smaller feature subsets and lower fitness values, indicating more efficient and effective feature selection. These findings demonstrate that RUS provides a robust balance between predictive performance and computational efficiency. Although the Ensemble-based methods yield competitive results in specific cases, they do not consistently outperform the RUS configuration and typically introduce additional computational cost due to increased sample size or multiple resampling stages. Overall, the comparative analysis confirms that BKOA-GOBL coupled with RUS represents the most effective configuration for addressing class imbalance within the tested fraud detection datasets.

**Table 6:** Outcomes of the suggested BKOA-GOBL under different resampling techniques in terms of the average classification accuracy, fitness, selected features, precision, recall, F-score and ROC_AUC.

**Measure**	**Datasets**	**RUS-*K*-NN**	**RUS-Xgb-tree**	**EasyEnsemble**	**BalancedBaggingClassifier**
	Australian	0.9051	**0.9135**	0.8406	0.8406
Accuracy	European	0.9479	0.9594	0.9862	**0.9883**
	CIC-MalMem-2022	0.9996	**0.9997**	0.9966	0.9976
	Real vs. Fake Job Postings Prediction	0.7412	0.8158	0.8405	**0.8440**
	**Ranking (*W*|*T*|*L*)**	0|0|4	2|0|2	0|0|4	2|0|2
Fitness	Australian	0.0984	**0.0910**	0.1588	0.1593
	European	0.0556	0.0446	0.0159	**0.0136**
	CIC-MalMem-2022	0.0027	**0.0021**	0.0055	0.0057
	Real vs. Fake Job Postings Prediction	0.2613	0.1874	**0.1627**	0.1636
	**Ranking (*W*|*T*|*L*)**	0|0|4	2|0|2	1|0|3	1|0|3
Features' Size	Australian	05.80	06.27	**01.30**	01.40
	European	11.17	13.13	06.70	**06.00**
	CIC-MalMem-2022	11.00	**09.10**	11.10	11.80
	Real vs. Fake Job Postings Prediction	727.8	709.8	**683.8**	695.6
	**Ranking (*W*|*T*|*L*)**	0|0|4	1|0|3	2|0|2	1|0|3
Precision	Australian	0.9033	**0.9053**	0.7377	0.7377
	European	**0.9755**	0.9751	0.9240	0.9573
	CIC-MalMem-2022	0.9995	**0.9996**	0.9955	0.9965
	Real vs. Fake Job Postings Prediction	0.7614	**0.8029**	0.7611	0.7902
	**Ranking (*W*|*T*|*L*)**	1|0|3	3|0|1	0|0|4	0|0|4
Recall	Australian	0.8320	0.8601	0.8824	**0.8850**
	European	0.9184	**0.9463**	0.7959	0.8059
	CIC-MalMem-2022	0.9996	**0.9997**	0.9976	0.9976
	Real vs. Fake Job Postings Prediction	0.6655	**0.8149**	0.7695	0.8014
	**Ranking (*W*|*T*|*L*)**	0|0|4	3|0|1	0|0|4	1|0|3
F1-Score	Australian	0.8660	**0.8795**	0.8036	0.8560
	European	0.9460	**0.9585**	0.9456	0.9118
	CIC-MalMem-2022	0.9996	**0.9997**	0.9965	0.9965
	Real vs. Fake Job Postings Prediction	0.7100	**0.8079**	0.5369	0.5435
	**Ranking (*W*|*T*|*L*)**	0|0|4	4|0|0	0|0|4	0|0|4
ROC_AUC	Australian	0.9107	**0.9162**	0.8492	0.8492
	European	0.9654	**0.9795**	0.8950	0.8949
	CIC-MalMem-2022	0.9998	**1.0000**	0.9966	0.9969
	Real vs. Fake Job Postings Prediction	0.7415	**0.8648**	0.6529	0.6565
	**Ranking (*W*|*T*|*L*)**	0|0|4	4|0|0	0|0|4	0|0|4

Bold values indicate the best-performing results for each evaluation measure.

The comparative findings demonstrate that ensemble-based resampling techniques can enhance minority-class sensitivity and classification stability, yet they do so at the expense of increased computational and memory complexity. Conversely, the RUS-based implementation of BKOA-GOBL presents a strategic compromise, delivering reliable performance with minimal preprocessing overhead.

This efficiency allows the proposed BKOA-GOBL framework to maintain scalability, fast convergence, and real-time applicability while preserving balanced FD performance. Therefore, the choice of RUS in this study reflects a deliberate trade-off between generalization and computational economy, aligning with the overarching goal of developing a robust and deployable FD system for big data financial environments. Future research directions may investigate hybrid resampling schemes that combine RUS with adaptive ensemble techniques to further improve detection sensitivity without sacrificing runtime efficiency.

### 4.6 Experimental outcomes of the proposed BKOA-GOBL vs. various recent MHTs employing *K*-NN classifier

Table 7 shows an evaluation of the proposed BKOA-GOBL algorithm performance via considerable MHTs utilizing a *K*-NN classifier regarding five benchmark datasets (Australian, European, Synthetic Financial Transaction Log, Real vs. Fake Job Postings Prediction, and CIC-MalMem-2022). Essential measures examined contain classification accuracy, fitness, selected features, precision, recall, F-score and ROC_AUC.

**Table 7:** Outcomes of the suggested BKOA-GOBL and various MHTs using the *K*-NN classifier concerning the average classification accuracy, fitness, selected features, precision, recall, F-score, and ROC_AUC.

**Measure**	**Datasets**	**Metric**	**BKOA-GOBL**	**BKOA**	**BMOA**	**BAVO**	**BSSO**	**BASO**	**BHGSO**	**BHHO**	**BSFO**	**BBA**	**BGOA**	**BPSO**
	Australian	Mean	**0.9051**	0.8935	0.8845	0.9024	0.9014	0.8780	0.8865	0.8986	0.8935	0.8870	0.9010	0.8944
		SD	**0.0022**	0.0065	0.0079	0.0036	0.0044	0.0075	0.0063	0.0059	0.0042	0.0091	0.0047	0.0055
	European	Mean	**0.9479**	0.9384	0.9315	0.9460	0.9450	0.9308	0.9342	0.9450	0.9372	0.9321	0.9418	0.9377
		SD	**0.0028**	0.0031	0.0064	0.0036	0.0035	0.0062	0.0035	0.0047	0.0033	0.0048	0.0031	0.0035
Accuracy	Synthetic financial transaction log	Mean	**0.9851**	**0.9851**	0.9849	**0.9851**	**0.9851**	0.9814	0.9850	**0.9851**	**0.9851**	0.9841	**0.9851**	**0.9851**
		SD	**0.0000**	**0.0000**	0.0003	**0.0000**	**0.0000**	0.0091	0.0002	**0.0000**	**0.0000**	0.0046	**0.0000**	**0.0000**
	CIC-MalMem-2022	Mean	**0.9996**	0.9993	0.9993	0.9995	0.9995	0.9994	0.9992	0.9995	0.9993	0.9992	0.9994	0.9993
		SD	**0.0002**	0.0004	0.0004	**0.0002**	**0.0002**	0.0003	0.0004	0.0003	**0.0002**	0.0004	0.0003	0.0003
	Real vs. Fake Job Postings Prediction	Mean	**0.7412**	0.7341	0.6926	0.7338	0.7279	0.7180	0.7136	0.7266	0.7239	0.7056	0.7324	0.7284
		SD	**0.0070**	0.0090	0.0408	0.0164	0.0243	0.0252	0.0299	0.0315	0.0215	0.0370	0.0173	0.0219
	**Ranking**	*W*|*T*|*L*	4|1|0	0|1|4	0|0|5	0|1|4	0|1|4	0|0|5	0|0|5	0|1|4	0|1|4	0|0|5	0|1|4	0|1|4
	Australian	Mean	**0.0984**	0.1105	0.1196	0.1011	0.1020	0.1261	0.1178	0.1051	0.1103	0.1169	0.1030	0.1095
		SD	**0.0024**	0.0069	0.0077	0.0034	0.0041	0.0072	0.0059	0.0058	0.0042	0.0087	0.0048	0.0057
	European	Mean	**0.0556**	0.0655	0.0725	0.0574	0.0587	0.0733	0.0703	0.0583	0.0666	0.0717	0.0620	0.0660
		SD	**0.0027**	0.0032	0.0063	0.0037	0.0034	0.0064	0.0035	0.0048	0.0028	0.0047	0.0031	0.0034
Fitness	Synthetic financial transaction log	Mean	**0.0177**	**0.0177**	0.0190	**0.0177**	**0.0177**	0.0230	0.0189	**0.0177**	**0.0177**	0.0197	**0.0177**	0.0179
		SD	**0.0000**	0.0003	0.0015	**0.0000**	**0.0000**	0.0101	0.0006	**0.0000**	**0.0000**	0.0050	**0.0000**	0.0005
	CIC-MalMem-2022	Mean	**0.0027**	0.0043	0.0044	**0.0027**	**0.0027**	0.0040	0.0049	0.0029	0.0040	0.0045	0.0038	0.0044
		SD	**0.0002**	0.0003	0.0004	0.0004	0.0005	0.0003	**0.0002**	0.0004	**0.0002**	0.0003	0.0004	**0.0002**
	Real vs. Fake Job Postings Prediction	Mean	**0.2613**	0.2682	0.3096	0.2688	0.2744	0.2842	0.2896	0.2759	0.2790	0.2964	0.2701	0.2740
		SD	**0.0070**	0.0089	0.0403	0.0161	0.0240	0.0249	0.0296	0.0313	0.0210	0.0368	0.0172	0.0217
	**Ranking**	*W*|*T*|*L*	3|2|0	0|1|4	0|0|5	0|2|3	0|2|3	0|0|5	0|0|5	0|1|4	0|1|4	0|0|5	0|1|4	0|0|5
	Australian	Mean	**05.80**	07.10	07.43	06.30	06.27	07.43	07.63	06.47	06.80	06.97	07.00	06.97
		SD	00.91	01.22	01.28	01.07	**00.73**	01.86	01.22	00.81	00.91	01.22	01.18	01.38
	European	Mean	**11.17**	13.67	13.97	11.90	12.67	14.40	15.37	11.60	13.33	13.57	13.10	13.00
		SD	02.41	02.44	02.81	**02.24**	03.12	03.19	02.44	02.67	02.81	02.65	02.49	02.32
Features' Size	Synthetic Financial Transaction Log	Mean	**02.00**	02.03	02.87	**02.00**	**02.00**	03.17	02.87	**02.00**	**02.00**	02.80	**02.00**	02.17
		SD	**00.00**	00.18	00.88	**00.00**	**00.00**	01.07	00.43	**00.00**	**00.00**	00.65	**00.00**	00.37
	CIC-MalMem-2022	Mean	**11.00**	18.87	19.13	11.27	11.77	17.57	21.37	12.67	17.37	19.33	16.57	19.60
		SD	02.03	02.22	02.51	02.08	02.38	01.82	02.24	02.44	**01.66**	02.44	02.36	01.98
	Real vs. Fake Job Postings Prediction	Mean	727.8	716.6	745.8	742.1	717.8	722.5	862.2	750.1	806.2	**705.2**	744.1	726.3
		SD	043.5	059.0	078.8	055.2	039.1	063.2	041.6	069.0	094.5	082.2	**019.4**	030.4
	**Ranking**	*W*|*T*|*L*	3|1|1	0|0|5	0|0|5	0|1|4	0|1|4	0|0|5	0|0|5	0|1|4	0|1|4	1|0|4	0|1|4	0|0|5
	Australian	Mean	**0.9033**	0.8767	0.8550	0.9029	0.8997	0.7722	0.8679	0.8896	0.8805	0.8630	0.8972	0.8814
		SD	0.0152	**0.0149**	0.0225	0.0193	0.0198	0.1514	0.0202	0.0232	0.0161	0.0264	0.0242	0.0183
	European	Mean	**0.9755**	0.9708	0.9646	0.9730	0.9743	0.8761	0.9675	0.9740	0.9732	0.9687	0.9718	0.9691
		SD	**0.0053**	0.0067	0.0087	0.0069	0.0066	0.1987	0.0068	0.0063	0.0065	0.0077	0.0072	0.0080
Precision	Synthetic Financial Transaction Log	Mean	**0.9856**	0.9749	0.9752	0.9749	0.9749	0.9749	0.9751	0.9749	0.9749	0.9742	0.9749	0.9749
		SD	0.1496	**0.0000**	0.0003	**0.0000**	**0.0000**	**0.0000**	0.0003	**0.0000**	**0.0000**	0.0049	**0.0000**	0.0001
	CIC-MalMem-2022	Mean	**0.9995**	0.9994	0.9994	**0.9995**	**0.9995**	0.9994	0.9993	**0.9995**	**0.9995**	0.9994	**0.9995**	0.9994
		SD	**0.0002**	0.0003	0.0003	**0.0002**	**0.0002**	**0.0002**	0.0003	**0.0002**	**0.0002**	0.0003	**0.0002**	0.0003
	Real vs. Fake Job Postings Prediction	Mean	**0.7614**	0.7514	0.7063	0.7504	0.7473	0.7040	0.7281	0.7430	0.7423	0.7241	0.7541	0.7472
		SD	**0.0104**	0.0185	0.0546	0.0268	0.0305	0.1282	0.0458	0.0419	0.0246	0.0484	0.0267	0.0312
	**Ranking**	*W*|*T*|*L*	4|1|0	0|0|5	0|0|5	0|1|4	0|1|4	0|0|5	0|0|5	0|1|4	0|1|4	0|0|5	0|1|4	0|0|5
	Australian	Mean	**0.8320**	0.8288	0.8294	0.8255	0.8261	0.8183	0.8183	0.8294	0.8242	0.8268	0.8281	0.8261
		SD	0.0149	0.0208	0.0254	0.0163	**0.0141**	0.0361	0.0208	0.0184	0.0186	0.0254	0.0180	0.0150
	European	Mean	**0.9184**	0.9034	0.8952	0.9170	0.9136	0.8925	0.8980	0.9139	0.8986	0.8925	0.9095	0.9037
		SD	0.0070	0.0078	0.0134	0.0094	0.0105	0.0101	0.0075	0.0094	**0.0064**	0.0101	0.0082	0.0086
Recall	Synthetic Financial Transaction Log	Mean	**0.9957**	**0.9957**	0.9951	**0.9957**	**0.9957**	0.9918	0.9954	**0.9957**	**0.9957**	0.9945	**0.9957**	**0.9957**
		SD	**0.0000**	**0.0000**	0.0008	**0.0000**	**0.0000**	0.0086	0.0005	**0.0000**	**0.0000**	0.0043	**0.0000**	0.0002
	CIC-MalMem-2022	Mean	**0.9996**	0.9992	0.9993	0.9995	0.9995	0.9993	0.9991	0.9995	0.9992	0.9990	0.9993	0.9993
		SD	**0.0003**	0.0006	0.0005	**0.0003**	**0.0003**	0.0005	0.0007	0.0004	0.0005	0.0006	0.0005	0.0006
	Real vs. Fake Job Postings Prediction	Mean	**0.6655**	0.6622	0.6151	0.6636	0.6504	0.6416	0.6463	0.6548	0.6444	0.6229	0.6525	0.6530
		SD	**0.0175**	0.0299	0.0717	0.0277	0.0552	0.0661	0.0709	0.0478	0.0424	0.0664	0.0217	0.0354
	**Ranking**	*W*|*T*|*L*	4|1|0	0|1|4	0|0|5	0|1|4	0|1|4	0|0|5	0|0|5	0|1|4	0|1|4	0|0|5	0|1|4	0|1|4
	Australian	Mean	**0.8660**	0.8518	0.8415	0.8621	0.8611	0.8320	0.8419	0.8580	0.8511	0.8439	0.8608	0.8526
		SD	**0.0036**	0.0100	0.0107	0.0043	0.0050	0.0118	0.0086	0.0074	0.0065	0.0122	0.0050	0.0071
	European	Mean	**0.9460**	0.9359	0.9285	0.9441	0.9429	0.9277	0.9314	0.9430	0.9344	0.9290	0.9396	0.9352
		SD	0.0037	0.0034	0.0070	0.0039	0.0039	0.0065	**0.0030**	0.0051	0.0035	0.0052	0.0034	0.0037
F1-Score	Synthetic Financial Transaction Log	Mean	**0.9852**	**0.9852**	0.9851	**0.9852**	**0.9852**	0.9815	**0.9852**	**0.9852**	**0.9852**	0.9843	**0.9852**	**0.9852**
		SD	**0.0000**	**0.0000**	0.0003	**0.0000**	**0.0000**	0.0090	0.0002	**0.0000**	**0.0000**	0.0046	**0.0000**	**0.0000**
	CIC-MalMem-2022	Mean	**0.9996**	0.9993	0.9993	0.9995	0.9995	0.9994	0.9992	0.9995	0.9993	0.9992	0.9994	0.9993
		SD	**0.0002**	0.0004	0.0004	**0.0002**	**0.0002**	0.0003	0.0004	0.0003	**0.0002**	0.0004	0.0003	0.0003
	Real vs. Fake Job Postings Prediction	Mean	**0.7100**	0.7032	0.6546	0.7036	0.6938	0.6827	0.6812	0.6948	0.6892	0.6672	0.6991	0.6959
		SD	**0.0098**	0.0133	0.0515	0.0161	0.0363	0.0412	0.0392	0.0377	0.0297	0.0480	0.0162	0.0231
	**Ranking**	*W*|*T*|*L*	4|1|0	0|1|4	0|0|5	0|1|4	0|1|4	0|0|5	0|1|4	0|1|4	0|1|4	0|0|5	0|1|4	0|1|4
	Australian	Mean	**0.9107**	0.9071	0.9011	0.9086	0.9083	0.8933	0.9000	0.9083	0.9044	0.9011	0.9091	0.9068
		SD	**0.0045**	0.0114	0.0132	0.0055	0.0068	0.0139	0.0136	0.0078	0.0122	0.0139	0.0088	0.0102
	European	Mean	**0.9654**	0.9576	0.9550	0.9636	0.9627	0.9566	0.9573	0.9631	0.9608	0.9567	0.9598	0.9600
		SD	**0.0067**	0.0072	0.0068	0.0074	**0.0067**	0.0073	**0.0067**	0.0071	0.0068	0.0089	0.0077	0.0070
ROC_AUC	Synthetic Financial Transaction Log	Mean	**0.9935**	0.9934	**0.9935**	0.9934	0.9934	0.9922	**0.9935**	0.9934	0.9934	0.9932	0.9934	0.9934
		SD	0.0002	**0.0000**	0.0002	**0.0000**	**0.0000**	0.0032	0.0002	0.0000	**0.0000**	0.0017	**0.0000**	0.0001
	CIC-MalMem-2022	Mean	**0.9998**	**0.9998**	**0.9998**	**0.9998**	**0.9998**	**0.9998**	**0.9998**	**0.9998**	**0.9998**	0.9997	**0.9998**	0.9997
		SD	**0.0001**	**0.0001**	**0.0001**	**0.0001**	**0.0001**	**0.0001**	0.0002	**0.0001**	**0.0001**	0.0002	**0.0001**	**0.0001**
	Real vs. Fake Job Postings Prediction	Mean	**0.7415**	0.7341	0.7082	0.7358	0.7298	0.7297	0.7223	0.7295	0.7341	0.7106	0.7359	0.7370
		SD	0.0129	**0.0123**	0.0417	0.0170	0.0240	0.0240	0.0276	0.0308	0.0200	0.0378	0.0200	0.0165
	**Ranking**	*W*|*T*|*L*	3|2|0	0|1|4	0|2|3	0|1|4	0|1|4	0|1|4	0|2|3	0|1|4	0|1|4	0|0|5	0|1|4	0|0|5

Bold values indicate the best-performing results for each evaluation measure.

Table 7 presents the outcomes of the BKOA-GOBL based on *K*-NN and its peers regarding classification accuracy across five datasets used (Australian, European, Synthetic Financial Transaction Log, Real vs. Fake Job Postings Prediction, and CIC-MalMem-2022). The performance of each algorithm is evaluated based on average accuracy and SD from multiple runs, shedding light on their reliability and effectiveness. The proposed BKOA-GOBL ranked first, achieving the highest average accuracy and smallest SD across all datasets, which reflects its stability and exceptional performance. For instance, in the Australian benchmark, BKOA-GOBL records a mean accuracy of 0.9051 with an SD of 0.0022, while on CIC-MalMem-2022, it achieves a perfect score of 0.9996 with an SD of just 0.0002. The BAVO comes closely behind, consistently ranked second in average accuracy across most datasets and providing competitive SD values, including an average accuracy of 0.9024 on the Australian dataset and 0.9460 on the European dataset.

In addition, the proposed BKOA-GOBL ranked first, achieving the highest average precision, recall, F1-measures, and smallest SD across most benchmark datasets, which reflects its stability and exceptional performance. The BAVO comes closely behind, consistently ranked second in average precision, recall, and F1 measures across most datasets and provides competitive SD values. These measures emphasize that the proposed BKOA-GOBL has a balanced performance and strong reliability. Precision demonstrates its success in minimizing false positives, while recall estimates its sensitivity to true positives. The F1-score presents a combined assessment of both precision and recall, reflecting overall classification quality. The consistently low SD values indicate BKOA-GOBL's stability and effectiveness across numerous runs, resulting in minimal variability and reduced risk of performance decline.

Moreover, the proposed BKOA-GOBL achieves the smallest feature reduction size across four of the five benchmark datasets, demonstrating its effectiveness in selecting the most appropriate attributes while ensuring high classification accuracy. BKOA-GOBL achieves the smallest mean feature size in Australian (5.80), European (11.17), Synthetic Financial Transaction Log (2.00), and CIC-MalMem-2022 (11.00), significantly reducing the size of the chosen attributes compared to other MHTs. The ranking demonstrates BKOA-GOBL's superiority with three wins, one tie, and one loss, making it one of the most effective techniques for FS across all benchmark datasets. Finally, the proposed BKOA-GOBL ranked first, achieving the smallest fitness values and smallest SD across all benchmark datasets, which reflects its stability and exceptional performance.

The ROC_AUC results further confirm the superiority and consistency of the proposed BKOA-GOBL among other competing MHTs across all benchmark datasets. It achieves the highest mean AUC with the lowest standard deviation in nearly all cases, demonstrating exceptional ability to distinguish fraudulent from legitimate instances under varying decision thresholds. For example, on the Synthetic Financial Transaction Log (0.9935) and CIC-MalMem-2022 (0.9998) datasets, BKOA-GOBL reaches near-perfect AUC values with extremely small variability, reflecting remarkable reliability and robustness. Even on the more challenging Real vs. Fake Job Postings dataset, it still secures the highest mean AUC while maintaining competitive SD values. Its low SD demonstrates strong reliability and minimal sensitivity to data variation. Competing algorithms such as BMOA and BAVO rank closely behind but consistently show higher variability, further reinforcing the strong stability and discriminative capability of the proposed approach. The ranking clearly confirms the superiority of BKOA-GOBL, achieving three wins, two ties, and no losses, positioning it as the most effective FS technique across all benchmark datasets.

### 4.7 Experimental outcomes of the proposed BKOA-GOBL vs. various recent MHTs employing Xgb-tree classifier

Table 8 shows an evaluation of the proposed BKOA-GOBL algorithm performance via considerable MHTs utilizing an Xgb-tree classifier regarding five benchmark datasets (Australian, European, Synthetic Financial Transaction Log, Real vs. Fake Job Postings Prediction, and CIC-MalMem-2022). Essential measures examined classification accuracy, fitness, selected features, precision, recall, F-score and ROC_AUC.

**Table 8:** Outcomes of the suggested BKOA-GOBL and various MHTs using Xgb-tree classifier concerning the average classification accuracy, fitness, selected features, precision, recall, F-score and ROC_AUC.

**Measure**	**Datasets**	**Metric**	**BKOA-GOBL**	**BKOA**	**BMOA**	**BAVO**	**BSSO**	**BASO**	**BHGSO**	**BHHO**	**BSFO**	**BBA**	**BGOA**	**BPSO**
	Australian	Mean	**0.9135**	0.8973	0.8833	0.9068	0.9053	0.8778	0.8870	0.9022	0.8949	0.8853	0.9027	0.8969
		SD	**0.0061**	0.0075	0.0094	**0.0061**	0.0075	0.0091	0.0078	0.0074	0.0079	0.0075	0.0072	0.0081
	European	Mean	**0.9594**	0.9526	0.9459	0.9582	0.9567	0.9489	0.9492	0.9548	0.9508	0.9479	0.9558	0.9525
		SD	**0.0023**	0.0044	0.0042	0.0039	0.0034	0.0043	0.0035	0.0040	0.0027	0.0051	0.0030	0.0033
Accuracy	Synthetic financial transaction log	Mean	**0.9917**	0.9915	0.9915	0.9915	0.9915	0.9915	0.9915	0.9915	0.9915	0.9915	0.9915	0.9915
		SD	0.0008	0.0002	**0.0000**	**0.0000**	**0.0000**	0.0013	0.0004	**0.0000**	**0.0000**	0.0012	**0.0000**	0.0002
	CIC-MalMem-2022	Mean	**0.9997**	0.9996	0.9996	**0.9997**	**0.9997**	0.9996	0.9996	**0.9997**	0.9996	0.9996	0.9996	0.9996
		SD	**0.0001**	0.0002	0.0003	**0.0001**	**0.0001**	0.0001	0.0002	**0.0001**	**0.0001**	0.0002	0.0002	0.0002
	Real vs. Fake Job Postings Prediction	Mean	**0.8158**	0.8002	0.7868	0.8097	0.8095	0.7908	0.7930	0.8111	0.7988	0.7907	0.8047	0.8003
		SD	**0.0060**	0.0073	0.0093	0.0074	0.0078	0.0061	0.0068	0.0081	0.0067	0.0088	**0.0060**	0.0084
	**Ranking**	*W*|*T*|*L*	4|1|0	0|0|5	0|0|5	0|1|4	0|1|4	0|0|5	0|0|5	0|1|4	0|0|5	0|0|5	0|0|5	0|0|5
	Australian	Mean	**0.0910**	0.1066	0.1206	0.0970	0.0984	0.1264	0.1179	0.1016	0.1094	0.1183	0.1011	0.1066
		SD	**0.0056**	0.0071	0.0093	0.0059	0.0071	0.0095	0.0078	0.0072	0.0075	0.0070	0.0070	0.0079
	European	Mean	**0.0446**	0.0516	0.0586	0.0457	0.0473	0.0555	0.0558	0.0494	0.0537	0.0563	0.0484	0.0518
		SD	**0.0020**	0.0043	0.0040	0.0039	0.0034	0.0039	0.0032	0.0039	**0.0020**	0.0048	0.0027	0.0033
Fitness	Synthetic financial transaction log	Mean	**0.0142**	**0.0142**	0.0148	**0.0142**	**0.0142**	0.0155	0.0143	**0.0142**	**0.0142**	0.0146	**0.0142**	**0.0142**
		SD	**0.0000**	0.0001	0.0010	**0.0000**	**0.0000**	0.0013	0.0003	**0.0000**	**0.0000**	0.0010	**0.0000**	0.0001
	CIC-MalMem-2022	Mean	**0.0021**	0.0037	0.0036	**0.0021**	**0.0021**	0.0034	0.0042	0.0024	0.0033	0.0037	0.0031	0.0038
		SD	**0.0002**	0.0003	0.0004	0.0004	0.0003	**0.0002**	**0.0002**	0.0004	**0.0002**	0.0004	0.0003	**0.0002**
	Real vs. Fake Job Postings Prediction	Mean	**0.1874**	0.2028	0.2166	0.1935	0.1937	0.2123	0.2109	0.1921	0.2052	0.2124	0.1986	0.2028
		SD	**0.0060**	0.0071	0.0091	0.0073	0.0078	**0.0060**	0.0066	0.0080	0.0066	0.0086	**0.0060**	0.0083
	**Ranking**	*W*|*T*|*L*	3|2|0	0|1|4	0|0|5	0|2|3	0|2|3	0|0|5	0|0|5	0|1|4	0|1|4	0|0|5	0|1|4	0|1|4
	Australian	Mean	**06.27**	07.00	07.20	06.57	06.47	07.53	08.33	06.60	07.47	06.63	06.57	06.50
		SD	01.86	01.83	01.90	01.80	01.77	02.26	**01.49**	01.99	01.67	01.54	01.50	01.67
	European	Mean	**13.13**	14.20	15.03	14.00	13.33	14.60	16.63	14.00	14.73	14.20	13.90	14.30
		SD	**01.78**	02.29	02.89	02.52	02.45	03.31	02.37	02.70	02.42	02.50	02.80	02.15
Features' size	Synthetic financial transaction log	Mean	**04.00**	04.07	04.67	**04.00**	**04.00**	04.90	04.30	**04.00**	**04.00**	04.33	**04.00**	04.07
		SD	**00.00**	00.25	00.70	**00.00**	**00.00**	00.87	00.46	**00.00**	**00.00**	00.54	**00.00**	00.25
	CIC-MalMem-2022	Mean	**09.10**	17.37	16.93	09.53	09.47	15.73	19.80	10.97	15.37	17.37	14.33	17.70
		SD	01.74	01.62	02.53	01.78	01.63	01.12	01.40	01.89	**00.88**	01.99	01.47	01.39
	Real vs. Fake Job Postings Prediction	Mean	**709.8**	718.6	789.6	730.3	723.5	730.5	855.0	730.7	846.0	735.6	750.3	727.2
		SD	**023.7**	036.6	084.2	051.6	047.4	075.4	039.2	041.7	063.5	080.9	046.8	036.8
	**Ranking**	*W*|*T*|*L*	4|1|0	0|0|5	0|0|5	0|1|4	0|1|4	0|0|5	0|0|5	0|1|4	0|1|4	0|0|5	0|1|4	0|0|5
	Australian	Mean	**0.9053**	0.8722	0.8629	0.8931	0.8849	0.7513	0.8659	0.8851	0.8769	0.8616	0.8850	0.8755
		SD	0.0269	0.0223	0.0241	0.0215	0.0236	0.1574	0.0211	**0.0189**	0.0246	0.0268	0.0226	0.0236
	European	Mean	**0.9751**	0.9668	0.9627	0.9692	0.9696	0.9737	0.9646	0.9696	0.9654	0.9641	0.9689	0.9668
		SD	**0.0060**	0.0074	0.0095	0.0094	0.0064	0.0433	0.0096	0.0079	0.0081	0.0080	0.0065	0.0088
Precision	Synthetic financial transaction log	Mean	**0.9884**	0.9880	0.9882	0.9879	0.9879	0.9879	0.9880	0.9879	0.9879	0.9879	0.9879	0.9880
		SD	0.0008	0.0004	0.0018	**0.0000**	**0.0000**	**0.0000**	0.0008	**0.0000**	**0.0000**	0.0021	**0.0000**	0.0004
	CIC-MalMem-2022	Mean	0.9996	0.9996	0.9996	0.9996	0.9996	**0.9997**	0.9996	0.9996	0.9996	0.9996	0.9996	0.9996
		SD	**0.0001**	**0.0001**	**0.0001**	**0.0001**	**0.0001**	**0.0001**	**0.0001**	**0.0001**	**0.0001**	**0.0001**	**0.0001**	**0.0001**
	Real vs. Fake Job Postings Prediction	Mean	**0.8029**	0.7855	0.7741	0.7921	0.7943	0.7166	0.7830	0.7972	0.7855	0.7762	0.7878	0.7867
		SD	**0.0117**	**0.0117**	0.0133	0.0138	0.0147	0.1424	0.0127	0.0137	0.0150	0.0148	0.0126	0.0122
	**Ranking**	*W*|*T*|*L*	4|0|1	0|0|5	0|0|5	0|0|5	0|0|5	1|0|4	0|0|5	0|0|5	0|0|5	0|0|5	0|0|5	0|0|5
	Australian	Mean	**0.8601**	0.8477	0.8150	0.8503	0.8562	0.8105	0.8222	0.8458	0.8340	0.8235	0.8477	0.8418
		SD	**0.0172**	0.0285	0.0302	**0.0172**	0.0211	0.0279	0.0208	0.0194	0.0219	0.0300	0.0213	0.0263
	European	Mean	**0.9463**	0.9371	0.9272	0.9363	0.9425	0.9303	0.9323	0.9388	0.9347	0.9299	0.9415	0.9367
		SD	0.0098	0.0099	0.0094	0.0098	0.0089	0.0127	0.0110	0.0099	0.0094	**0.0082**	0.0095	0.0103
Recall	Synthetic financial transaction log	Mean	**0.9954**	0.9951	0.9952	0.9951	0.9951	0.9951	0.9951	0.9951	0.9951	0.9951	0.9951	0.9951
		SD	**0.0000**	**0.0000**	0.0004	**0.0000**	**0.0000**	**0.0000**	0.0001	**0.0000**	**0.0000**	0.0005	**0.0000**	**0.0000**
	CIC-MalMem-2022	Mean	**0.9997**	0.9996	0.9996	**0.9997**	**0.9997**	0.9996	0.9996	**0.9997**	**0.9997**	0.9996	**0.9997**	0.9996
		SD	**0.0002**	0.0003	0.0005	0.0003	**0.0002**	0.0003	0.0004	0.0003	0.0003	0.0003	0.0003	0.0004
	Real vs. Fake Job Postings Prediction	Mean	**0.8149**	0.7993	0.7806	**0.8149**	0.8104	0.7915	0.7830	0.8102	0.7955	0.7884	0.8083	0.7974
		SD	0.0180	0.0203	0.0186	0.0200	0.0181	0.0256	0.0207	0.0192	0.0236	0.0214	0.0199	**0.0152**
	**Ranking**	*W*|*T*|*L*	3|2|0	0|0|5	0|0|5	0|2|3	0|1|4	0|0|5	0|0|5	0|1|4	0|1|4	0|0|5	0|1|4	0|0|5
	Australian	Mean	**0.8795**	0.8591	0.8376	0.8708	0.8699	0.8305	0.8431	0.8647	0.8544	0.8413	0.8655	0.8577
		SD	**0.0070**	0.0109	0.0140	0.0078	0.0099	0.0128	0.0108	0.0104	0.0101	0.0104	0.0096	0.0114
	European	Mean	**0.9585**	0.9516	0.9446	0.9575	0.9558	0.9477	0.9481	0.9539	0.9497	0.9467	0.9550	0.9514
		SD	**0.0024**	0.0046	0.0043	0.0040	0.0036	0.0047	0.0037	0.0042	0.0028	0.0052	0.0032	0.0035
F1-Score	Synthetic financial transaction log	Mean	0.9915	0.9915	**0.9918**	0.9915	0.9915	0.9915	0.9917	0.9915	0.9915	0.9915	0.9915	0.9915
		SD	**0.0000**	0.0002	0.0008	**0.0000**	**0.0000**	0.0013	0.0004	**0.0000**	**0.0000**	0.0012	**0.0000**	0.0002
	CIC-MalMem-2022	Mean	**0.9997**	0.9996	0.9996	**0.9997**	**0.9997**	0.9996	0.9996	**0.9997**	0.9996	0.9996	0.9996	0.9996
		SD	**0.0001**	0.0002	0.0003	**0.0001**	**0.0001**	**0.0001**	0.0002	**0.0001**	**0.0001**	0.0002	0.0002	0.0002
	Real vs. Fake Job Postings Prediction	Mean	**0.8079**	0.7921	0.7772	0.8031	0.8020	0.7827	0.7827	0.8034	0.7901	0.7820	0.7976	0.7919
		SD	**0.0068**	0.0088	0.0103	0.0082	0.0081	0.0084	0.0084	0.0088	0.0083	0.0100	0.0072	0.0089
	**Ranking**	*W*|*T*|*L*	3|1|1	0|0|5	1|0|4	0|1|4	0|1|4	0|0|5	0|0|5	0|1|4	0|0|5	0|0|5	0|0|5	0|0|5
	Australian	Mean	**0.9162**	0.9056	0.9061	0.9132	0.9122	0.9010	0.9034	0.9099	0.9063	0.9070	0.9070	0.9071
		SD	**0.0050**	0.0101	0.0113	0.0051	0.0085	0.0104	0.0090	0.0089	0.0106	0.0116	0.0121	0.0108
	European	Mean	**0.9795**	0.9772	0.9774	0.9789	0.9780	0.9757	0.9771	0.9780	0.9765	0.9755	0.9795	0.9777
		SD	0.0053	0.0046	0.0043	0.0045	0.004	0.0049	0.0049	0.0044	0.0047	0.0052	0.0042	**0.0038**
ROC_AUC	Synthetic financial transaction log	Mean	**0.9990**	0.9986	0.9989	0.9985	0.9985	**0.9990**	0.9987	0.9985	0.9985	0.9987	0.9985	0.9986
		SD	0.0004	0.0002	0.0004	**0.0000**	**0.0000**	0.0004	0.0003	**0.0000**	**0.0000**	0.0004	**0.0000**	0.0002
	CIC-MalMem-2022	Mean	**1.0000**	0.9999	**1.0000**	0.9999	**1.0000**	**1.0000**	0.9999	0.9999	0.9999	0.9999	0.9999	0.9999
		SD	**0.0001**	**0.0001**	**0.0001**	**0.0001**	**0.0001**	**0.0001**	**0.0001**	**0.0001**	**0.0001**	**0.0001**	**0.0001**	**0.0001**
	Real vs. Fake Job Postings Prediction	Mean	**0.8648**	0.8576	0.8490	0.8602	0.8586	0.8488	0.8493	0.8612	0.8552	0.8436	0.8575	0.8509
		SD	0.0113	0.0124	0.0106	**0.0093**	0.0103	0.0134	0.0118	0.0135	0.0119	0.0175	0.0113	0.0120
	**Ranking**	*W*|*T*|*L*	3|2|0	0|0|5	0|1|4	0|0|5	0|1|4	0|2|3	0|0|5	0|0|5	0|0|5	0|0|5	0|0|5	0|0|5

Bold values indicate the best-performing results for each evaluation measure.

Table 8 presents the outcomes of the BKOA-GOBL based on Xgb-tree and its peers concerning classification accuracy across five utilized datasets (Australian, European, Synthetic Financial Transaction Log, Real vs. Fake Job Postings Prediction, and CIC-MalMem-2022). The performance of each algorithm is evaluated based on average accuracy and SD from multiple runs, shedding light on their reliability and effectiveness. The proposed BKOA-GOBL with Xgb-tree ranked first, achieving the highest average accuracy and smallest SD across all datasets, which reflects its stability and exceptional performance. For instance, in the Australian benchmark, BKOA-GOBL records a mean accuracy of 0.9135 with an SD of 0.0061, while on CIC-MalMem-2022, it achieves a perfect score of 0.9997 with an SD of just 0.0001. The BAVO comes closely behind, consistently ranked second in average accuracy across most datasets and providing competitive SD values, including an average accuracy of 0.0.9582 on the Australian dataset and 0.9460 on the European dataset.

In addition, the proposed BKOA-GOBL with Xgb-tree ranked first, achieving the highest average precision, recall, F1-measures, and smallest SD across most benchmark datasets, which reflects its stability and exceptional performance. The BAVO comes closely behind, consistently ranked second in average precision, recall, and F1 measures across most datasets and provides competitive SD values. These measures emphasize that the proposed BKOA-GOBL with Xgb-tree has a balanced performance and strong reliability. Precision demonstrates its success in minimizing false positives, while recall estimates its sensitivity to true positives. The F1-score presents a combined assessment of both precision and recall, reflecting overall classification quality. The consistently low SD values indicate that BKOA-GOBL exhibits stability and effectiveness, as demonstrated by numerous runs, resulting in minimal variability and reduced risk of performance decline.

Moreover, the proposed BKOA-GOBL with Xgb-tree achieves the smallest size of feature reduction in all benchmark datasets, establishing its effectiveness in selecting the most appropriate attributes while ensuring high classification accuracy. BKOA-GOBL with Xgb-tree obtains the smallest mean size of features in Australian (06.27), European (13.13), Synthetic Financial Transaction Log (04.00), Real vs. Fake Job Postings Prediction (709.8), and CIC-MalMem-2022 (09.10), significantly decreasing the size of chosen attributes compared to other MHTs. The ranking demonstrates BKOA-GOBL's superiority over Xgb-tree, with four wins, one tie, and zero losses, making it one of the most effective techniques for FS across all benchmark datasets. Finally, the proposed BKOA-GOBL with Xgb-tree ranked first, achieving the smallest fitness values and smallest SD across all benchmark datasets, which reflects its stability, exceptional performance, and balancing capability between accuracy and number of selected features.

Regarding the ROC_AUC results, the proposed BKOA-GOBL with Xgb-tree consistently achieves the highest ROC_AUC scores and the lowest SD across most benchmark datasets, reflecting its strong ability to distinguish fraudulent from legitimate instances under varying threshold settings. For example, it secures an almost perfect AUC of 1.0000 with an SD of 0.0001 on the CIC-MalMem-2022 dataset and records leading performance across the Synthetic Financial Transaction Log (0.9990) and European (0.9795) datasets as well. Competing algorithms rank noticeably lower, with fewer wins and higher variability, reinforcing the superior and stable discriminative power of the BKOA-GOBL with XGB-Tree classifier. The ranking clearly confirms the superiority of BKOA-GOBL, achieving three wins, two ties, and no losses, positioning it as the most effective FS technique across all benchmark datasets.

### 4.8 Convergence investigation

**Figure 2:**
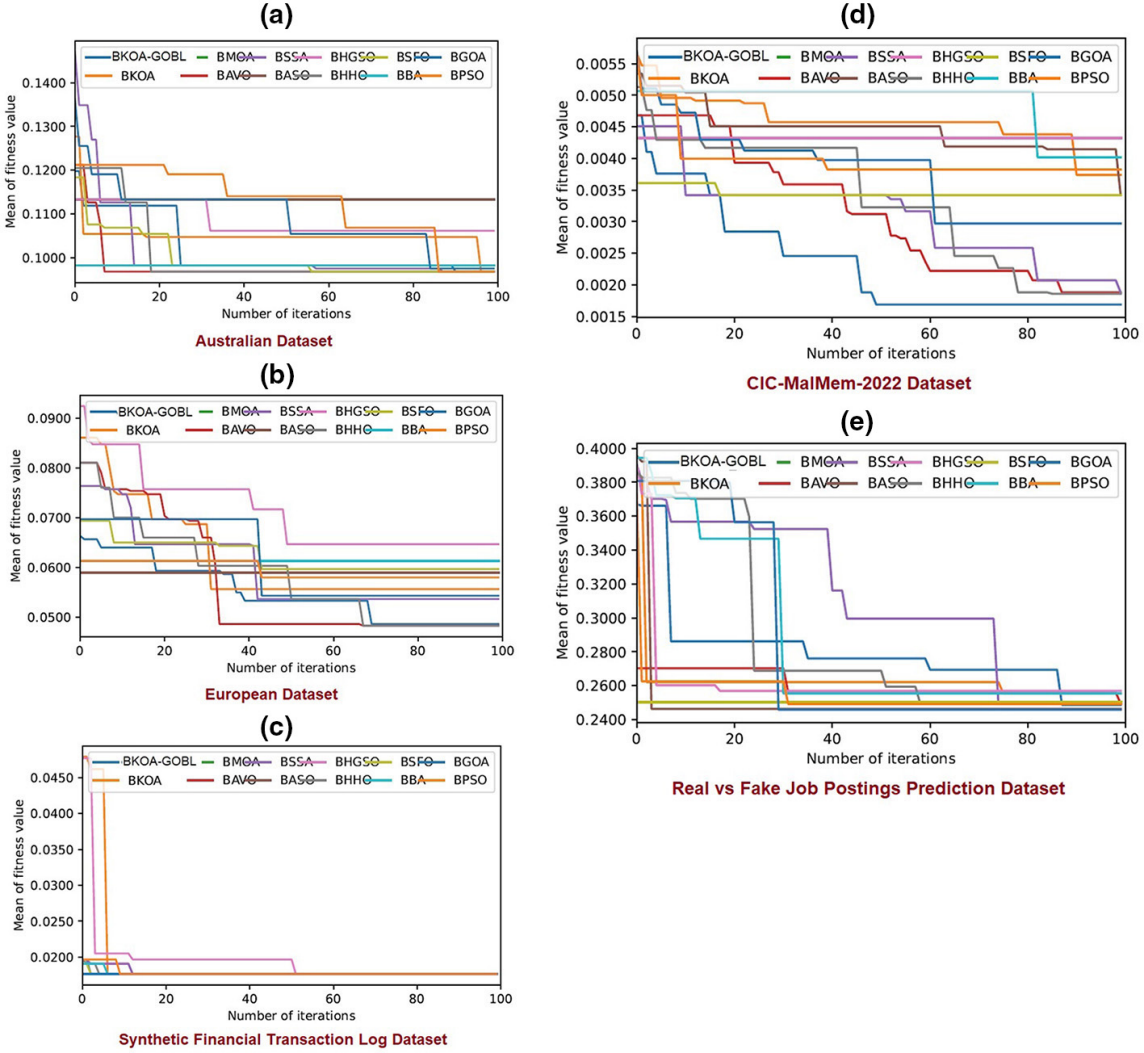
Convergence curves of the suggested BKOA-GOBL and its peers concerning the *K*-NN classifier via the datasets: **(a)** Australian, **(b)** European, **(c)** Synthetic Financial Transaction Log, **(d)** CIC-MalMem-2022, and **(e)** Real vs Fake Job Postings Prediction.

**Figure 3:**
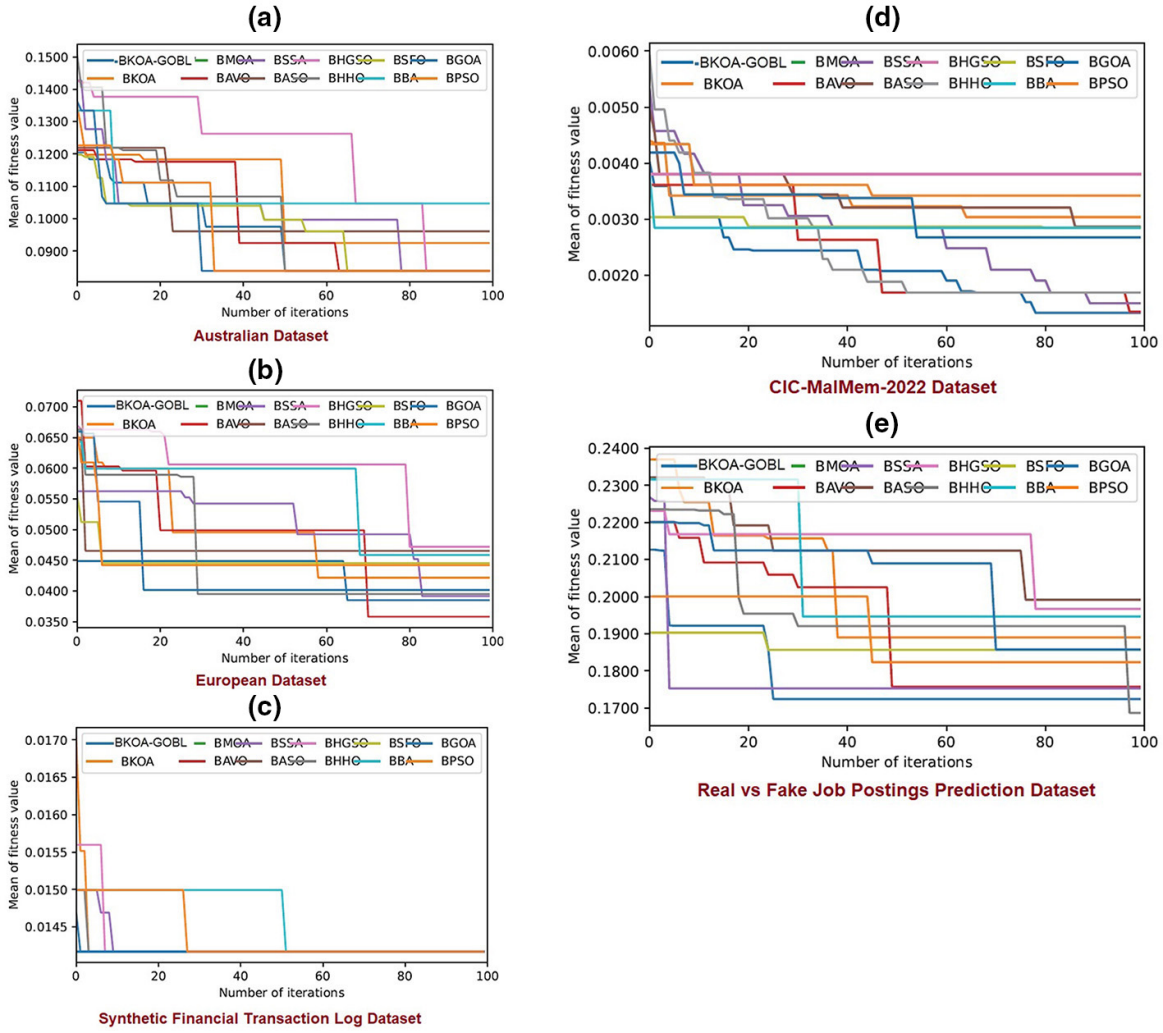
Convergence curves of the suggested BKOA-GOBL and its peers concerning the Xgb-tree classifier via the datasets: **(a)** Australian, **(b)** European, **(c)** Synthetic Financial Transaction Log, **(d)** CIC-MalMem-2022, and **(e)** Real vs Fake Job Postings Prediction.

The asymptotic capabilities of the proposed approaches (BKOA-GOBL with *k*-NN and BKOA-GOBL with Xgb-tree) are examined in this section for addressing fraud and malware classification using five datasets. The aim is to evaluate the performance of convergence, as shown in Figures 2, 3. These figures demonstrate that the suggested BKOA-GOBL with *K*-NN and Xgb-tree classifiers achieves both optimal and rapid convergence with all datasets, outperforming other MHTs under the same conditions of population size and number of iterations.

### 4.9 Precision-recall analysis

**Figure 4:**
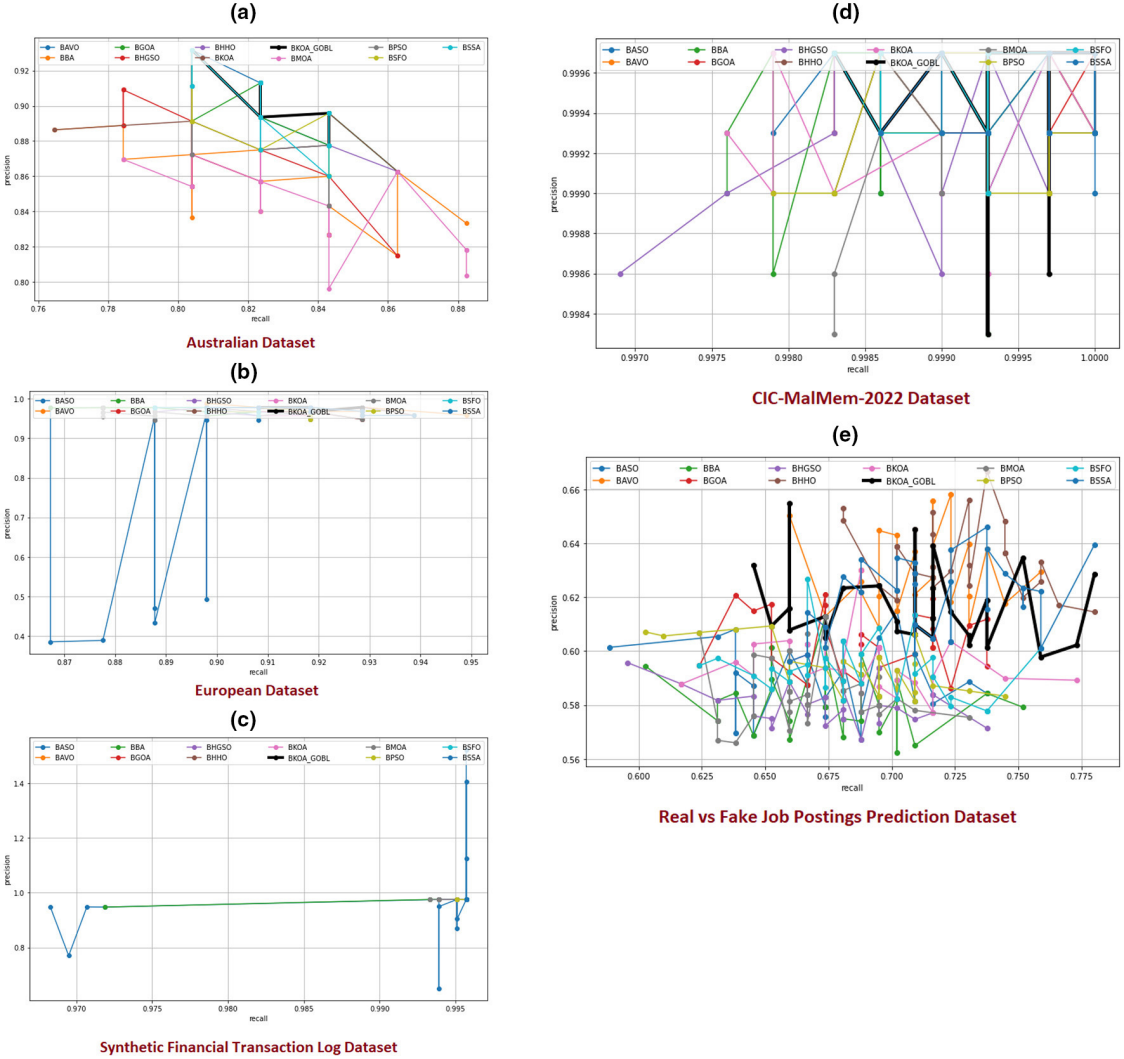
Precision-Recall curves of the suggested BKOA-GOBL and its peers concerning the *K*-NN classifier via the datasets: **(a)** Australian, **(b)** European, **(c)** Synthetic Financial Transaction Log, **(d)** CIC-MalMem-2022, and **(e)** Real vs Fake Job Postings Prediction.

**Figure 5:**
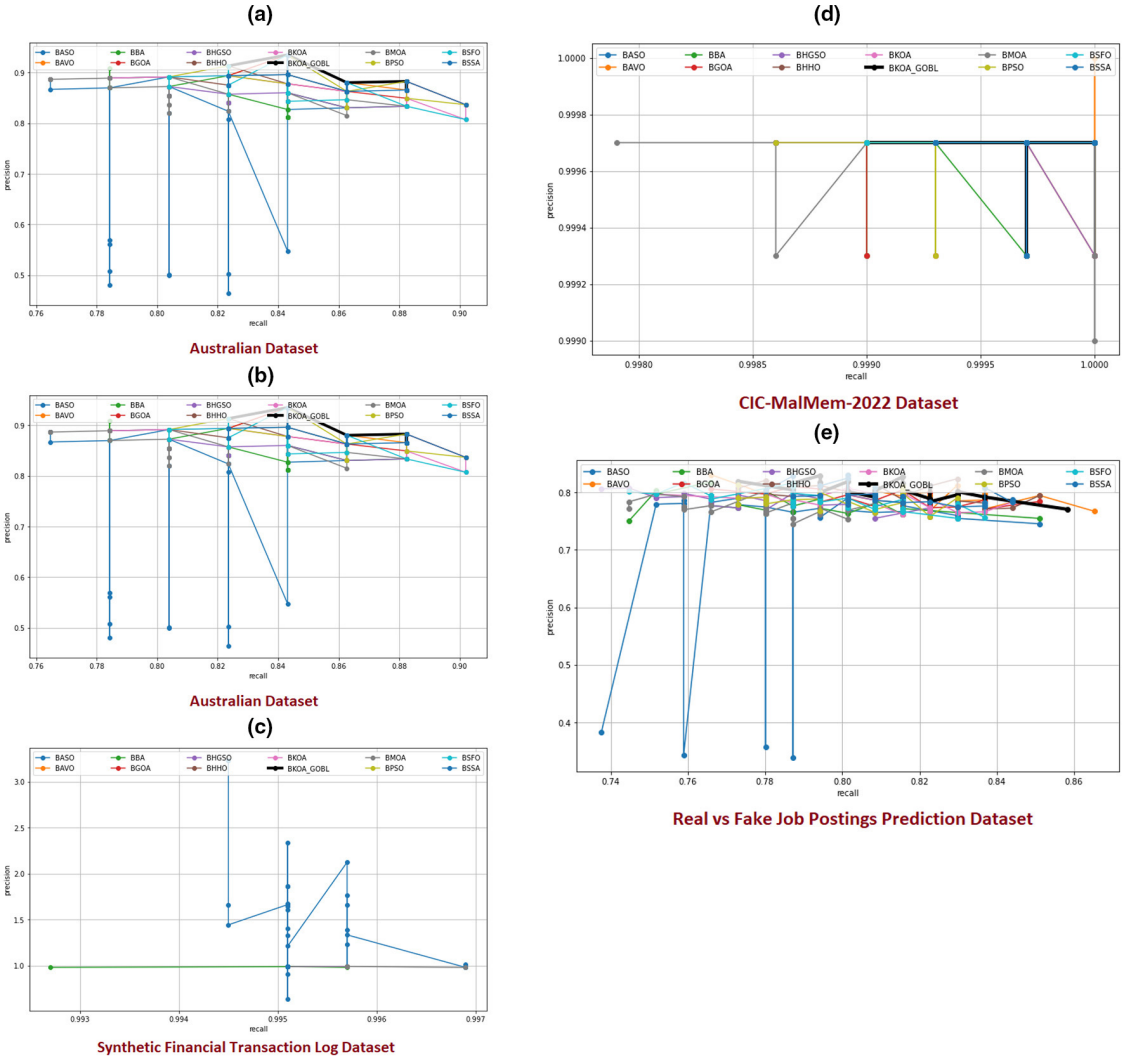
Precision-Recall curves of the suggested BKOA-GOBL and its peers concerning the Xgb-tree classifier via the datasets: **(a)** Australian, **(b)** European, **(c)** Synthetic Financial Transaction Log, **(d)** CIC-MalMem-2022, and **(e)** Real vs Fake Job Postings Prediction.

The precision-recall curves provide a detailed view of the classification performance of the proposed BKOA-GOBL framework under varying discrimination thresholds. Unlike accuracy, which can be misleading in highly imbalanced datasets, precision-recall curves focus on two critical measures for FD–precision (the ability to avoid false alarms) and recall (the ability to detect true fraud). As shown in Figures 4, 5, the superiority of the suggested BKOA-GOBL with *K*-NN and Xgb-tree classifiers is especially pronounced with most datasets, where most alternative approaches show sharp declines in precision as recall increases. The consistently smooth and high-positioned curves reinforce that the suggested BKOA-GOBL effectively avoids local optima and yields reliable features that distinguish fraudulent patterns even under difficult data conditions.

### 4.10 Wilcoxon's rank-sum test

The Wilcoxon signed-rank test was used to perform a statistical analysis comparing the fitness function values from the BKOA-GOBL and other algorithms, as shown in Tables 9, 10 (Derrac et al., 2011). The aim was to determine if there were any significant differences between them.

**Table 9:** Wilcoxon's test for the average classification error of the proposed BKOA-GOBL and its peers concerning *K*-NN.

**BKOA-GOBL-*k*-NN vs**.	***R* ^+^**	***R* ^−^**	***P*-value**	**Confidence interval**	**Exact confidence**	**Winner**
BKOA	10.0	0.0	0.04461	0, 0.0116	0.9375	BKOA-GOBL
BMOA	15.0	0.0	0.030971	0.0002, 0.0346	0.9375	BKOA-GOBL
BAVO	10.0	0.0	0.04461	0, 0.00505	0.9375	BKOA-GOBL
BSSO	10.0	0.0	0.04461	0, 0.0085	0.9375	BKOA-GOBL
BASO	15.0	0.0	0.030971	0.0002, 0.0271	0.9375	BKOA-GOBL
BHGSO	15.0	0.0	0.030971	0.0001, 0.0231	0.9375	BKOA-GOBL
BHHO	10.0	0.0	0.04461	0, 0.01055	0.9375	BKOA-GOBL
BSFO	10.0	0.0	0.04461	0, 0.01445	0.9375	BKOA-GOBL
BBA	15.0	0.0	0.030971	0.0004, 0.02685	0.9375	BKOA-GOBL
BGOA	10.0	0.0	0.04461	0, 0.00745	0.9375	BKOA-GOBL
BPSO	10.0	0.0	0.04461	0, 0.01175	0.9375	BKOA-GOBL

**Table 10:** Wilcoxon's test for the average classification error of the proposed BKOA-GOBL and its peers concerning Xgb-tree.

**BKOA-GOBL with Xgb-tree vs**.	***R* ^+^**	***R* ^−^**	***P*-value**	**Confidence interval**	**Exact confidence**	**Winner**
BKOA	15.0	0.0	0.030971	0, 0.0116	0.9375	BKOA-GOBL
BMOA	15.0	0.0	0.030971	0.0002, 0.0346	0.9375	BKOA-GOBL
BAVO	10.0	0.0	0.04461	0, 0.00505	0.9375	BKOA-GOBL
BSSO	10.0	0.0	0.04461	0, 0.0085	0.9375	BKOA-GOBL
BASO	15.0	0.0	0.030971	0.0002, 0.0271	0.9375	BKOA-GOBL
BHGSO	15.0	0.0	0.030971	0.0001, 0.0231	0.9375	BKOA-GOBL
BHHO	10.0	0.0	0.04461	0, 0.01055	0.9375	BKOA-GOBL
BSFO	15.0	0.0	0.06250	0, 0.01445	0.9375	BKOA-GOBL
BBA	15.0	0.0	0.030971	0.0004, 0.02685	0.9375	BKOA-GOBL
BGOA	15.0	0.0	0.030971	0, 0.00745	0.9375	BKOA-GOBL
BPSO	15.0	0.0	0.030971	0, 0.01175	0.9375	BKOA-GOBL

The Wilcoxon signed-rank test is a non-parametric method used in hypothesis testing to compare two related samples. It involves calculating the differences between paired results for a set of problems and ranking these differences by their absolute values. The process then computes the totals of ranks for positive differences (*R*^+^) and negative differences (*R*^−^), identifying the smaller of the two. The significance of the test is determined using a *p*-value; if it is below 0.05, it indicates that the differences between the two approaches are statistically significant, suggesting strong evidence against the null hypothesis.

The analysis of the results in Tables 9, 10 demonstrates that the BKOA-GOBL method significantly outperforms other methods when implemented with either *k*-NN or Xgb-tree classifiers across the entire test scenarios. The *p*-values in the tables are consistently below the 0.05 threshold, indicating that the enhancements provided by BKOA-GOBL are statistically significant and not merely coincidental. These results confirm the superior performance of the BKOA-GOBL method compared to other alternatives.

In summary, the results of the Wilcoxon test showcase the strong performance of the BKOA-GOBL algorithm, reaffirming its statistical superiority. The consistent rejection of the null hypothesis indicates that the enhancements made by BKOA-GOBL are significant and worthwhile.

### 4.11 Real-time integration feasibility

The BKOA-GOBL framework shows real promise for real-time FD, particularly in environments where decisions must be made in milliseconds. By reducing the number of features, the entire process is accelerated–less data means faster scoring and fewer chances of system slowdowns. That's a significant win when you're trying to catch fraud before a transaction is processed. These improvements make it a strong candidate for integration into live monitoring systems, where speed and accuracy are non-negotiable.

Reducing the feature set doesn't just help with speed–it also reduces memory usage, which is a significant advantage when deploying models in production. Whether it's running on a cloud server or a small device at the edge, like a payment terminal, leaner models are easier to manage. The beauty of BKOA-GOBL lies in its flexibility: for smaller workloads, it runs smoothly on regular CPUs, but when dealing with massive volumes–such as in an extensive financial network–it can scale up with GPUs or distributed systems to maintain speed and responsiveness.

Another strength of BKOA-GOBL is its ability to work in both batch and streaming setups. Batch processing is ideal for retraining and updating the model periodically, while streaming enables real-time decisions as transactions occur. Although this study didn't simulate live data streams directly, the performance gains we observed suggest that the system is well-equipped for such an environment. Testing it on actual transaction flows would be a logical next step–and one that could really show how well it holds up under pressure.

## 5 Practical deployment considerations

Although the proposed BKOA-GOBL has demonstrated significant efficacy in benchmark datasets, transitioning from experimental validation to deployment in real-world financial systems necessitates addressing multiple operational and systemic challenges. This section outlines the key considerations for effective integration.

### 5.1 Integration into financial infrastructure

The implementation of the proposed BKOA-GOBL system within current financial ecosystems necessitates the seamless incorporation with extant legacy systems, the establishment of secure data transmission channels, and adherence to prevailing regulatory frameworks. Principal challenges associated with integration encompass:

- The capability to achieve seamless integration and compatibility among a variety of data formats and sources across different institutional frameworks.
- Tuning the algorithm for low-latency environments where FD must occur instantly.
- Maintaining data confidentiality and adhering to standards.

### 5.2 Scalability and computational efficiency

Financial institutions process vast volumes of transactions daily. BKOA-GOBL must scale efficiently to handle:

- Optimizing parallel processing and memory usage for large-scale deployment.
- Leveraging distributed architectures to support scalable and resilient operations.
- Automating updates to maintain performance as fraud patterns evolve.

### 5.3 Interpretability and regulatory compliance

In FD, interpretability is crucial for establishing trust, ensuring auditability, and maintaining legal accountability. To meet these needs:

- Providing clear insights into which features influenced detection decisions.
- Integrating post-hoc interpretability methods such as Explainable AI to visualize decision boundaries and model behavior.
- Allowing analysts to validate and override automated decisions when necessary.

### 5.4 Operational monitoring and maintenance

Long-term success of BKOA-GOBL depends on robust operational support:

- Monitoring for changes in data distribution that may degrade model performance.
- Prioritizing alerts to reduce false positives and analyst fatigue.
- Incorporating user feedback to refine model accuracy and relevance.

## 6 Conclusion and future directions

This study introduced a robust and adaptive FD methodology, BKOA-GOBL, for improved FD and convergence behavior. The method effectively balances exploration and exploitation through planetary motion-inspired dynamics and addresses class imbalance using RUS. Two classifiers, *K*-NN and Xgb-tree, were employed to assess the classification accuracy of selected feature subsets. Comprehensive experiments across five diverse and real-world datasets demonstrated that BKOA-GOBL consistently outperforms traditional classifiers and twelve state-of-the-art MHAs in terms of several performance indicators, such as accuracy, feature reduction, and fitness. Specifically, the proposed methodology achieved classification accuracies of up to 99.96% and feature reduction rates of up to 81.82%, while maintaining high precision, recall, and F1-scores (all exceeding 0.95) across the datasets. The BKOA-GOBL exhibited superior exploration and exploitation compared to its counterparts. The statistical significance of its superiority was confirmed using Wilcoxon's rank-sum test at a 5% significance level. These results affirm the proposed model's adaptability, efficiency, and robustness, making it a promising tool for real-world FD applications in high-dimensional and imbalanced data environments. The proposed BKOA-GOBL, while effective, has several limitations: the use of RUS helps balance the dataset but may remove valuable information and reduce classification accuracy; the integration of BKOA and mutation strategies enhances FS efficiency but introduces additional computational complexity compared to simpler models; its success depends heavily on optimal parameter tuning, requiring extra effort in hyperparameter optimization; and although validated on five benchmark datasets, its applicability to real-time, large-scale transaction data across diverse regions and industries remains to be investigated.

Looking ahead, future research can focus on enhancing the capabilities of BKOA-GOBL through hybridization with other swarm-based or evolutionary algorithms to improve its global search ability and convergence behavior. Exploring the adaptation of BKOA-GOBL to real-time FD systems using streaming data environments, where latency and adaptability are critical. Incorporating online learning mechanisms into the framework would allow it to update dynamically as new transaction patterns emerge, enhancing its responsiveness to evolving fraud tactics. The integration of BKOA-GOBL with advanced classification techniques, such as DL and neural networks, may yield further improvements. Furthermore, exploring multi-objective extensions of BKOA-GOBL could allow simultaneous optimization of multiple conflicting goals, such as maximizing accuracy while minimizing computational cost or energy consumption. These directions offer valuable opportunities to evolve BKOA-GOBL into a more powerful and versatile optimization framework.

## Data Availability

The original contributions presented in the study are included in the article/supplementary material, further inquiries can be directed to the corresponding authors.
